# Whole Mitochondrial Genome Detection and Analysis of Two- to Four-Generation Maternal Pedigrees Using a New Massively Parallel Sequencing Panel

**DOI:** 10.3390/genes14040912

**Published:** 2023-04-14

**Authors:** Dan Peng, Jiaojiao Geng, Jingyi Yang, Jiajun Liu, Nana Wang, Riga Wu, Hongyu Sun

**Affiliations:** 1 Faculty of Forensic Medicine, Zhongshan School of Medicine, Sun Yat-sen University, Guangzhou 510080, Chinawurg3@mail.sysu.edu.cn (R.W.); 2 Guangdong Province Translational Forensic Medicine Engineering Technology Research Center, Sun Yat-sen University, Guangzhou 510080, China

**Keywords:** mitochondrial DNA, ForenSeq mtDNA Whole Genome Kit, massively parallel sequencing, heteroplasmy, maternal pedigree

## Abstract

Mitochondrial DNA (mtDNA) is an effective genetic marker in forensic practice, especially for aged bones and hair shafts. Detection of the whole mitochondrial genome (mtGenome) using traditional Sanger-type sequencing is laborious and time-consuming. Additionally, its ability to distinguish point heteroplasmy (PHP) and length heteroplasmy (LHP) is limited. The application of massively parallel sequencing in mtDNA detection helps researchers to study the mtGenome in-depth. The ForenSeq mtDNA Whole Genome Kit, which contains a total of 245 short amplicons, is one of the multiplex library preparation kits for the mtGenome. We used this system to detect the mtGenome in the blood samples and hair shafts of thirty-three individuals from eight two-generation pedigrees, one three-generation pedigree, and one four-generation pedigree. High-quality sequencing results were obtained. Ten unique mtGenome haplotypes were observed in the mothers from the ten pedigrees. A total of 26 PHPs were observed using the interpretation threshold of 6%. Eleven types of LHPs in six regions were evaluated in detail. When considering homoplasmic variants only, consistent mtGenome haplotypes were observed between the twice-sequenced libraries and between the blood and hair shafts from the same individual and among maternal relatives in the pedigrees. Four inherited PHPs were observed, and the remainder were de novo/disappearing PHPs in the pedigrees. Our results demonstrate the effective capability of the ForenSeq mtDNA Whole Genome Kit to generate the complete mtGenome in blood and hair shafts, as well as the complexity of mtDNA haplotype comparisons between different types of maternal relatives when heteroplasmy is considered.

## 1. Introduction

Circular double-stranded mitochondrial DNA (mtDNA) has been commonly detected in forensic cases where the samples are aged or lack nuclear DNA (e.g., rootless hair shafts or aged bones) [1,2]. It is also frequently used in genealogy, archaeology, evolutionary anthropology, and medical genetics [3,4]. As the mtDNA haplotypes are theoretically the same among individuals from the same maternal lineage, mtDNA is often used in maternal familial investigations [5,6] and for tracing the ancestry of remains [7]. However, when using the traditional Sanger-type sequencing, the detection of the whole mitochondrial genome (mtGenome) is laborious and time-consuming; thus, only hypervariable regions are usually detected in forensic practice. Additionally, the comparison guidelines were constructed for the control region only [8]. However, several studies have demonstrated that the variants in the coding region of the mtGenome can improve the discrimination power and haplogroup estimation [2,9,10,11]. Moreover, limited by the sensitivity of electrophoretograms, a minor allele frequency (MAF) of 15~20% has typically been used for point heteroplasmy (PHP) calls [11,12]. Additionally, distinguishing length heteroplasmy (LHP) in the homopolymeric stretches (C-stretches) often fails due to the fluorescence detection nature of Sanger-type sequencing.

At present, massively parallel sequencing (MPS), whereby a large number of markers and samples can be detected in one sequencing run [13,14], provides a new platform for mtGenome typing. As one of the first forensic genetic markers evaluated using MPS, mtDNA has been studied in populations worldwide [9,10,11,15,16] and in various tissues [17,18,19,20,21] through a variety of MPS multiplexes of the control region and mtGenome. In addition, the MAF of PHP calls ranges from 1% to 10% [22,23,24,25,26], even dropping to as low as 0.15% [27]. Successful detection of C-stretches has also been realized [28,29]. Studies evaluating the germline bottleneck size [30,31] and mtDNA transmissions in pedigrees [32,33] and those distinguishing between the mother and offspring [24] have also been carried out with MPS detection of the mtGenome.

Both long-amplicon multiplexes (usually divided into two ~8 kb amplicons or one ~9 kb amplicon plus one ~11 kb amplicon) [34,35,36] and short-amplicon multiplexes (e.g., the Precision ID mtDNA Whole Genome Panel) [10,37] have been established for the mtDNA mtGenome. Long amplicons can decrease the risk of contamination of nuclear mitochondrial DNA reads (NUMTs) and amplification failure caused by SNPs in the primer-binding region, while the requirement for the input genome DNA (gDNA) is relatively high (usually 1 ng) [34]. In addition, long amplicons are hard to amplify due to the homopolymeric stretches, and the fragmentation processes prior to sequencing increase the time of manual operation [9]. On the contrary, short-amplicon multiplexes require a relatively low quantity of input gDNA (usually 100 pg), while the NUMT contamination is non-negligible [38,39]. In addition, short-amplicon multiplexes are more suitable for degraded samples, and no extra fragmentation process is required as the amplicon size is suitable for the length of the sequencing reads. The fire-new ForenSeq mtDNA Whole Genome Kit (abbr. as ForenSeq mtGenome Kit below, Verogen, San Diego, CA, USA) [40] uses a 2-PCR approach, wherein small overlapping amplicons (60–209 bp, with a mean of 131 bp) are generated to achieve detection coverage of the whole mtGenome using a small amount of input DNA (20 pg to 100 pg). A total of 245 amplicons from 663 primers are enriched in 2-tiled primer mixes (with a 17 bp overlap on average, and a 3 bp overlap minimum). Additionally, the ForenSeq Universal Analysis Software v2.1 (abbr. as ForenSeq UAS v2.1 below, Verogen, San Diego, CA, USA) can automatically generate mtDNA variant calls that follow the nomenclature of the Scientific Working Group on DNA Analysis Methods (SWGDAM) [8] after being compared with the revised Cambridge Reference Sequence (rCRS). Meanwhile, an EMPOP-required format file [41] can be exported for further use.

The present study aimed to evaluate the performance of the ForenSeq mtGenome Kit in the detection of the mtGenome in blood samples and hair shafts, the ability of this panel to distinguish heteroplasmy, and the characteristics of variant transmission and variant difference between maternal relatives. Thus, we used this well-validated [42] multiplex to detect the blood samples and hair shafts of thirty-three individuals from two- to four-generation pedigrees. The sequencing results of the blood samples and hair shafts from the same individual and the concordance between the twice-sequenced libraries were evaluated. We presented the PHPs and LHPs in detail. Additionally, the variant transmission between mother and offspring pairs and variant differences between the individuals from ten more maternal relatives were also estimated.

## 2. Materials and Methods

### 2.1. Materials and Samples

All samples were anonymously collected from Guangdong Han Chinese people with informed consent. This study was approved by the Ethics Committee of Zhongshan School of Medicine, Sun Yat-sen University (No. [2020]044).

Both peripheral blood samples and hair shaft samples were collected from 11 individuals in a 4-generation pedigree, and peripheral blood samples were collected from 6 individuals in a 3-generation pedigree and 16 individuals in 8 2-generation pedigrees (the family trees for the 4- and 3-generation pedigrees are presented in Supplementary Figure S1). In total, 44 samples from 10 pedigrees were obtained, which included 33 blood samples and 11 hair shafts. Blood was collected using an EDTA anticoagulant tube or sterilized filter, and rootless hair shafts were obtained by cutting 0.5 cm above the scalps of the individuals.

### 2.2. DNA Extraction and Quantification

Prior to DNA extraction, the hair shafts were cleaned with 1% SDS, 5%(W/V) NaClO, sterilized distilled water, 10% ethanol, and 100% ethanol in turn [43]. The first 2 cm hair shaft from each individual was used for the following steps. The genomic DNA of the hair shafts or blood samples was extracted using the QIAamp DNA Investigator Kit [44] (Qiagen, Hilden, NRW, Germany; cat#56504) following the manufacturer’s instructions and was quantified with a Qubit 3.0 fluorometer using the Qubit dsDNA HS Assay Kit (Invitrogen, Eugene, OR, USA). Nuclease-free water was utilized as a negative extraction control during the DNA extraction methods (NC-EXH represents the negative extraction control for hair shaft extraction; NC-EXB represents the negative extraction control for blood sample extraction).

### 2.3. Library Preparation and Sequencing

DNA libraries were constructed using the ForenSeq mtGenome Kit according to the manufacturer’s instructions [45]. Briefly, the gDNA sample HL60 (Millipore-Sigma, St. Louis, MO, USA) was used as a positive amplification control, and nuclease-free water (provided in the ForenSeq mtGenome Kit) was used as a negative amplification control (NC-AMP). Thus, a total of 48 libraries were constructed. Then, the libraries were purified once and normalized using the bead-based method according to the instructions. A total of 16 normalized libraries were pooled together, and 5 μL of the pooled libraries was denatured, diluted, and finally added to the Miseq FGx Reagent Kit cartridge (Illumina, San Diego, CA, USA) for the first sequencing run, as recommended, to undergo paired-end sequencing-by-synthesis reactions using the Miseq FGx instrument (Illumina, San Diego, CA, USA). Additionally, the number of pooled libraries and volume of the loaded dilution in the following four runs were adjusted dynamically according to the previous sequencing quality (Table 1). Sixteen libraries were sequenced twice for the concordance study, resulting in a total of sixty-four sequenced libraries. Strict cleaning and separation methods were used to control and avoid contamination throughout the experiment according to the recommendations of the International Society of Forensic Genetics (ISFG) and SWGDAM [8,46,47].

### 2.4. Variant Calling and Data Analysis

The raw sequencing data were first analyzed using ForenSeq UAS v2.1 [48]. The sequencing quality metrics and total depth of each sample were obtained directly from the software. The read depth at each position was obtained using the VCF file. The strand bias, which measures the balance between forward and reverse reads at a particular position, was calculated as follows: 1− (read depth of the direction with a smaller number of reads/read depth of the direction with a larger number of reads). A strand bias value of 0 indicates no strand bias, and 1.0 indicates the presence of reads in only 1 direction [37]. The mtGenome coverage of a sample was calculated as the count of the genotyped nucleotide position divided by 16,569 (the expected total position). The relative read depth (RRD) was calculated as the read depth of the negative control divided by the read depth of the positive control to evaluate the contamination level of the negative controls.

The NUMTs and byproducts were removed automatically using UAS v2.1, and the sequence strings with mixed bases were screened with BLAST tools (https://blast.ncbi.nlm.nih.gov/, accessed on 20 December 2022). For variant calling, the Verogen mtDNA whole-genome analysis method (default thresholds) [42] was used, i.e., a variant call is supported when it meets or exceeds the analytical threshold (AT, 6%), interpretation threshold (IT, 6%), minimum Q-score (Q-score = 30), and minimum read count (reads = 45). The frequency of a variant refers to the total number of reads for the particular variant call divided by the total number of reads at that nucleotide position. 

The variants called using UAS v2.1 were checked using the ‘Alignment’ function on the EMPOP website [41], wherein SAM 2.0 was used on the basis of 5440 haplogroup motifs (PhyloTree, Build 17 [49]) following the phylogenetic concept and the recommendations of the ISFG [46,47]. A total of 2 C-stretch-related variants that deviated from the nomenclature were corrected manually as follows: (1) 16189c should be reported as 16189C and 16193c; (2) 310Y should be reported as 309.1, 309.2, etc., depending on the number of insertions in the read for this region [42]. All unexpected variants and heteroplasmies were checked using the Integrative Genomics Viewer (IGV) [50]. Haplogroups of mtDNA haplotypes were assigned using the ‘Haplogrouping’ function on the EMPOP website [41]. The variant concordance between the twice-sequenced libraries was also evaluated. The pairwise difference in coverage, the pairwise difference in the total depth, and the pairwise difference in the variant frequency were measured as the targeted data of the second sequencing run minus those of the first sequencing run. Pictures were generated using Excel and the ‘ggplot2′ package in the R software.

### 2.5. Variant Transmission and Variant Differences in Maternal Pedigrees

The variant transmission was evaluated between mother and offspring pairs, and the variant differences were evaluated between individuals from all maternal relatives. To classify the transmission of heteroplasmy between relatives, the types of heteroplasmy were defined as follows: inherited heteroplasmy was heteroplasmy that was observed in both the mother and her offspring; de novo heteroplasmy was heteroplasmy that was observed only in the offspring and absent in the mother; disappearing heteroplasmy was heteroplasmy that was only observed in the mother and was absent in her offspring [33]. The MAF change during transmission was estimated using the MAF of the later generation minus the MAF of the former generation [33]. Considering the fact that various types of tissues may be used in forensic genetics applications, the variants in blood samples and hair shafts from the two maternal relatives were cross-compared in pairs.

## 3. Results

### 3.1. Sequencing Overview

**Figure 1:**
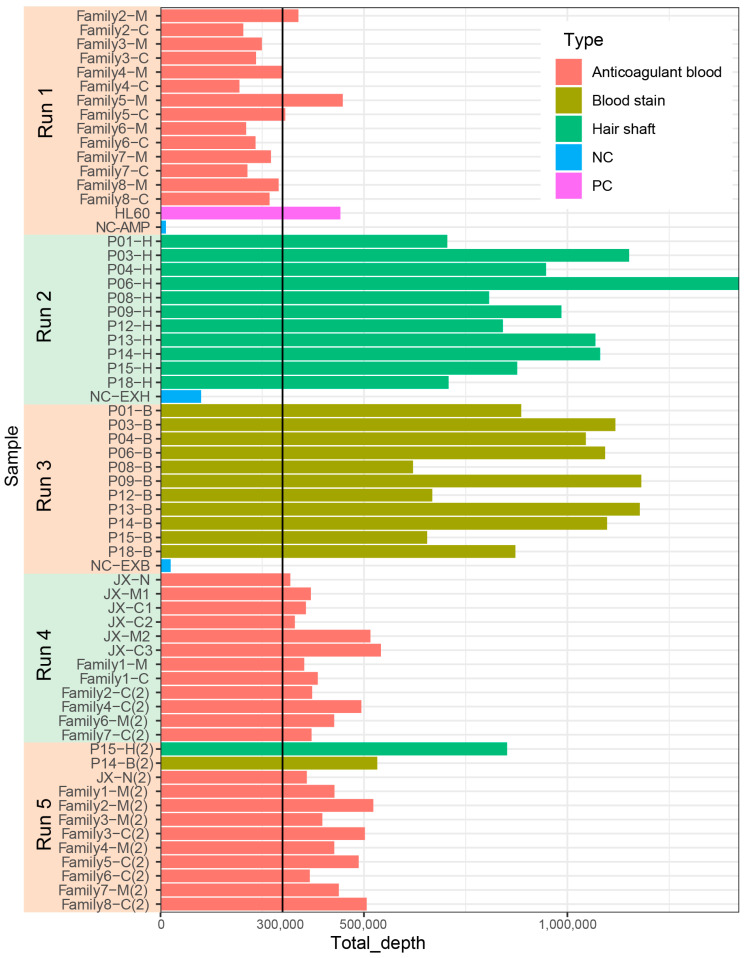
Total read depths of the sequenced libraries. ‘M’ represents mother in the family pair, ‘C’ represents offspring in the family pair, ‘H’ represents hair shaft, and ‘B’ represents blood sample. Sample IDs with ‘(2)’ correspond to the twice-sequenced libraries. The vertical ‘300,000′ line is the threshold of total read depth recommended by the manufacturer as a guarantee of complete mtGenome coverage.

**Figure 2:**
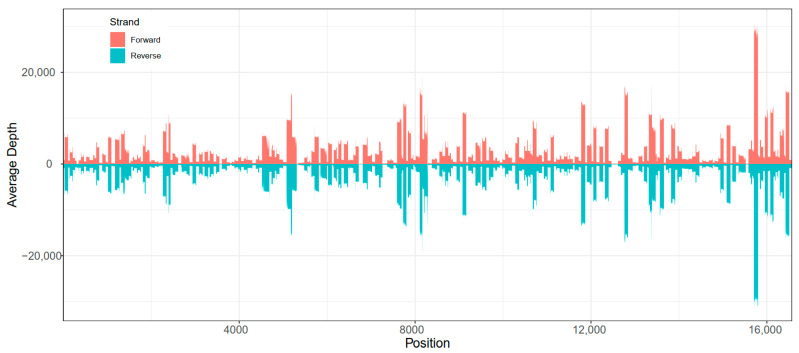
The average read depths of both forward and reverse strands at each nucleotide position in the mtGenome. Data from the 4 controls were not included, resulting in a total of 60 calculated datasets.

A total of 64 libraries (4 of which were controls) were sequenced in this study, in which 16 libraries were sequenced twice. The average cluster density was 1560.6 ± 228.32 k/mm^2^, and the average total read depth was 562,592 ± 331,109×, for all sequenced libraries (Table 1, Figure 1). The average total read depths for all anticoagulant blood samples, all blood stain samples, and all hair shafts were 362,102 ± 99,978×, 912,202 ± 228,578×, and 953,650 ± 196,052×, respectively. As shown in Figure 2, relatively low average read depths were mainly observed in the nucleotide positions (nps) 303–347, 3550–3606, 5307–5347, 6718–6810, 12,466–12,614, and 15,519–15,581. The average read depths of the control region and 37 gene-coding regions are shown in Table 2. The mtGenome coverage of the 60 target libraries ranged from 97.37% to 100% (with an average of 99.61% ± 0.60%).

**Figure 3:**
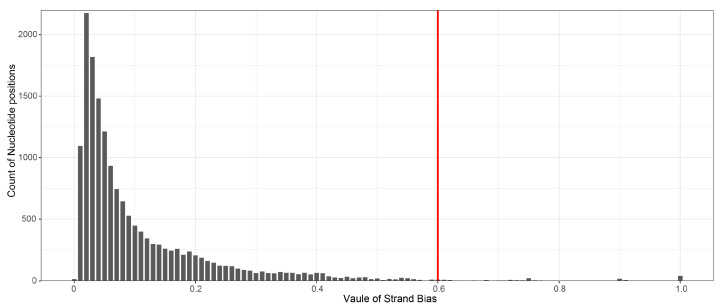
Overall strand bias distribution in the 60 detected sample libraries. A value of 0 indicates no strand bias, and 1.0 indicates the presence of reads in only 1 direction. Data from the 4 controls were not included, resulting in a total of 60 calculated datasets.

In the results of the 60 sample libraries, only 1.68% of all the positions had a strand bias value over 0.6 (Figure 3). Most of the strand bias values were equal to 1.0 at nps 303~347, which indicated only 1 direction of the sequencing reads. In addition, high strand biases were also observed at nps 1003–1018, 1095–1176, 1495–1546, 2262–2266, 2684–2694, 4565–4665, 6142–6149, 6784–6810, 7560–7590, 7960–7985, and 13,496–13,513.

A total of 34 variants were observed in HL60 using the default analysis thresholds, with an average read depth of 4107.34 ± 3378.87×. Among the observed variants, 33 were SNPs, and one was an insertion (315.1C). No base call was observed at np 6734. The haplogroup of HL60 used was assigned as J2b1a1a via ‘Haplogrouping’ tool. As for the negative control, no base call or variants were called in NC-AMP and NC-EXB using the default thresholds, while 22 variants were called in NC-EXH, with an average read depth of 627.68 ± 464.62×. The average RRDs of NC-AMP and HL60, NC-EXB and HL60, and NC-EXH and HL60 were 0.07%, 0.03%, and 5.40%, respectively (Supplementary Figure S2).

Among the 16 twice-sequenced libraries, both the total read depth and mtGenome coverage in the second sequencing run were higher than those in the corresponding libraries in the first sequencing run, except for P15-H and P14-B (Supplementary Figure S3). The pairwise depth differences were 40,738×~267,576× in the libraries, and the pairwise coverage differences were 0.07~1.94% (Supplementary Table S1). Overall, except for the two libraries from P14-B, the read depth and coverage in the second run were higher than those in the first for all the twice-sequenced libraries. However, when comparing the observed variants between the two sequencing results, differences occurred only in the C-stretch regions (see Supplementary Table S1 for details). The results of the better-performing libraries were used for the following analysis.

**Figure 4:**
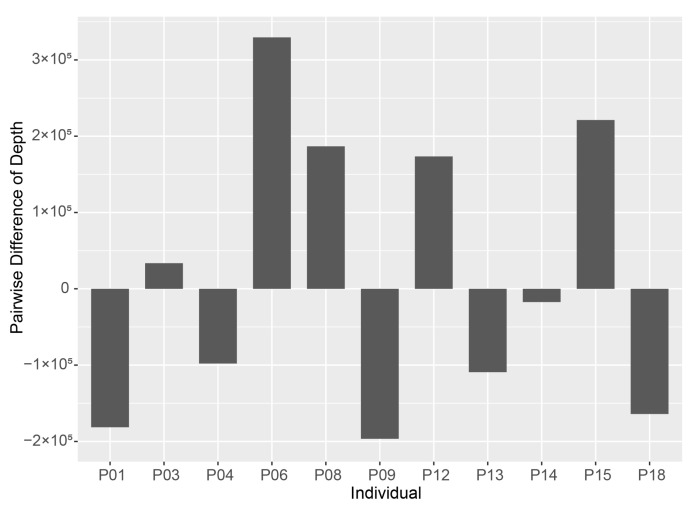
The pairwise differences in total depth between blood sample and hair shaft of the same individual. Total pairwise depth difference: the total depth of the hair shaft minus the total depth of the corresponding blood sample.

As for the 11 hair shafts and the corresponding blood samples from the same individuals, the total read depth did not show a significant tendency towards either of the tissues (Figure 4), and the mtGenome coverages were all 100%, except for P15-H (99.67%; no base call was reported at 54 nucleotide positions).

### 3.2. mtDNA Variant Polymorphism

**Figure 5:**
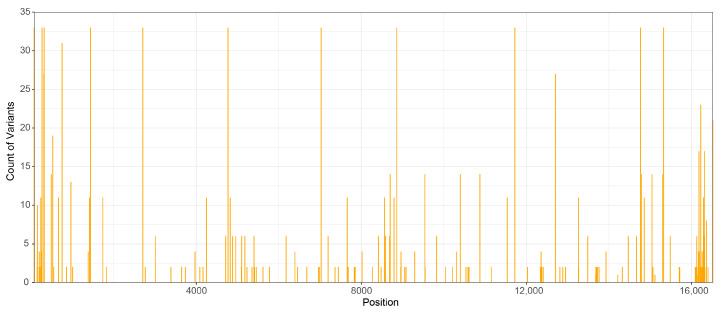
Variant distribution in the mtGenome. The maximum count is 33.

In the blood samples from 33 individuals, a total of 1247 variants were observed at 172 nucleotide positions, and 178 types of variants were included. Of the 178 types of variants, 167 were SNPs, 8 were insertions, and 3 were deletions (Supplementary Table S2). A total of 396 variants (31.76%; 56 types) were observed in the control region, 849 variants (68.08%; 121 types) were observed in the coding region, and 2 variants (1 type) were observed in the non-coding region (Table 2). The largest number of variants were observed in the 12S RNA (RNR1) and cytochrome b (CYB) gene-coding regions, with 136 and 132 variants, respectively. The variant distribution at each nucleotide position is presented in Figure 5. Variants 73G, 263G, 315.1C, 1438G, 2706G, 4769G, 7028T, 8860G, 11719A, 14766T, and 15326G were observed in all samples.

**Figure 6:**
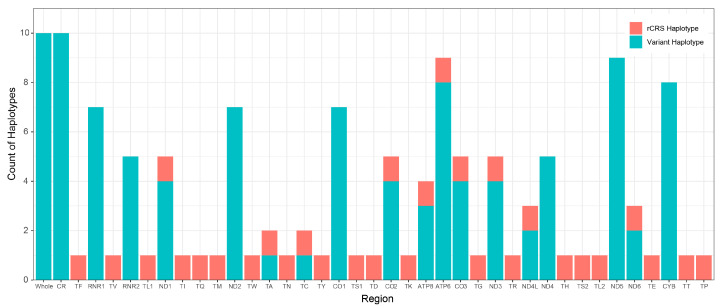
Count of haplotype types observed in the whole mtGenome, control region, and coding region.

Ten different mtDNA haplotypes were observed in the mothers from ten pedigrees in the control region, coding region, and whole mtGenome. Inside the coding region, different haplotypes were observed in 17 gene-coding regions, while the remaining 20 regions showed the same haplotypes. The top three haplotype types were observed in NADH dehydrogenase 5 (ND5), ATP synthase 6 (ATP6), and CYB, with nine, eight, and eight haplotypes, respectively (Figure 6).

The tri-alleles T9824A/C and C13683A/G were observed in this study, wherein variant call A was observed in four samples and variant call C was observed in two samples at np 9824, and variant calls A and G were observed in two samples, separately, at np13683. A total of 10 unique haplogroups were assigned in the 10 pedigrees, of which 5 were nested in super haplogroup M and 5 were nested in super haplogroup N. The assigned haplogroups were the same among the family members from the same maternal pedigrees (Supplementary Table S2).

### 3.3. Heteroplasmy

Among the 33 blood samples, 12 (36.36%) samples were observed to have 1~2 PHPs (14 PHPs in total; Supplementary Figure S4), wherein 10 were a mixed base of C and T (Y), 2 were a mixed base of A and C (M), 1 was a mixed base of A and G (R), and 1 was a mixed base of C and G (S). Four PHPs were observed in the control region, while the remaining ten were observed in the coding region. The MAFs of these PHPs were 6.39% ~ 35.95%. In the 11 hair samples, 7 (77.78%) samples were observed to have 1~3 PHPs (12 in total; Supplementary Figure S4), wherein 3 were a mixed base of C and T (Y), 2 were a mixed base of A and C (M), 6 were a mixed base of A and G (R), and 1 was a mixed base of C and G (S). One PHP was observed in the control region, while the remaining ten were observed in the coding region. The MAFs ranged from 6.97% to 49.09% (Table 3).

The mtDNA region of the ND5 gene contained the largest number of PHPs (four in the blood samples and three in the hair shafts) in both the blood samples and the hair shafts. In the 11 pairs of same-origin blood samples and hair shafts, no overlapping PHPs were observed in any tissue pairs.

Length variations that were different from the rCRS were observed at nps 303–315, 955–966, 12,417–12,426, 16,180–16,194, 249, 514–524, and 8271–8279. Sequence variations in the first four regions were caused by base insertions of C or A (np 12,425), while variations in the last three regions were due to base or fragment deletions. Excepting np 249 (249DEL), LHPs were observed in all the regions described above (Table 4).

To locate the accurate position of the poly-C or poly-A stretches, we took the non-repetitive bases at both ends of a fragment as the anchor position to grab the target fragment and used an abbreviated form to show the variant. For example, variant type ‘A-7C-T-6C-G’ represents sequence ‘ACCCCCCCTCCCCCCG’ from nps 302 to 316, wherein the consecutive Cs are interrupted by 310T, and compared with the rCRS, the variant of this fragment is 315.1C. The 315.1C variant was observed in all 44 samples, except for 1 sample that presented no base call in this region. Additionally, more than 7 Cs were observed at nps 303–309 in 35 samples. No transition of T > C was observed at np 310. LHP was observed when eight consecutive Cs existed. As shown in Table 4, the ‘A-8C-T-6C-G’ variant type was related to two haplotypes (309.1C and 309.1c), wherein the difference occurred at the frequency (or read count) of the reads with eight Cs.

The T961C variant led to a sequence with more than 10 consecutive Cs at nps 955–966. A total of 20 samples showed a sequence with 12 consecutive Cs varying in length, and 4 samples were observed to have a sequence with 13 consecutive Cs. Additionally, it is worth mentioning that the read count dropped precipitously at np 964, whose average read count was only 21.97% ± 5.23% of that at np 963. Similarly, the length variation at nps 16,180–16,194 was caused by T16189C, and the T > C transition at nps 16,182 and 16,183 exacerbated the C-stretches. As the only polyadenine stretch, LHP happened when nine consecutive As occurred at np 12425. Mixed variation in 523A, 524C, and 523–524DEL (with an average frequency of 84.52% ± 0.98%) was observed at nps 523–524. A similar situation was also observed for the 9bp deletion fragment at nps 8272–8280 (Table 4). The average frequency of deletion at nps 8275–8280 was 88.20% ± 2.52%, which led to a mixture of base calls and deletions under the default thresholds.

### 3.4. Variant Transmission between Mother and Offspring Pairs

A total of 53 comparisons were carried out for the 23 mother–offspring pairs (Table 5), in which 23 comparisons were between blood samples of the two members, 10 comparisons were between hair shafts of the two members, 10 comparisons were between the mother’s blood sample and the offspring’s hair shaft, and 10 comparisons were between the mother’s hair shaft and the offspring’s blood sample. Both homoplasmic variants and heteroplasmic variants were compared. As T961C was related to the LHPs at nps 956–965, the different variants at np 961 were incorporated within the LHPs at nps 956–965, and the differences were counted once in this region. The mtDNA haplotypes were completely the same in 11 comparisons, and consistent haplotypes were observed in 49 comparisons when heteroplasmic variants were ignored. All homoplasmic variants were transmitted from the mother to her offspring in both the blood samples and hair shafts, except the hair shaft of P06 (P06-H), in which a de novo 4475C (located in the ND2-coding region) was observed. Thus, the haplotype of P06-H was different from the haplotypes of their mother and her offspring. 

**Figure 7:**
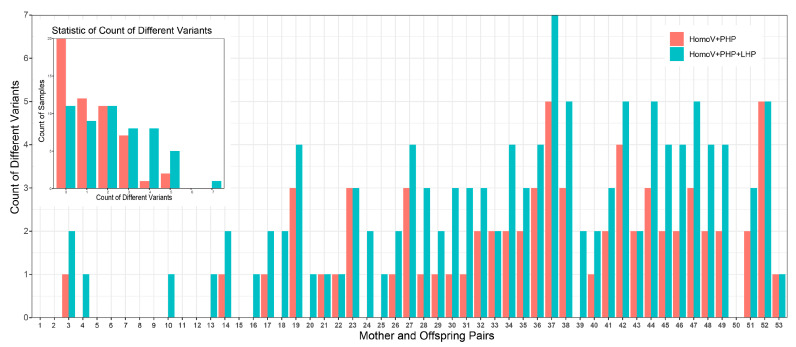
Counts of different variants in all compared mother–offspring pairs.

When heteroplasmic variants were considered, 42 comparisons showed different haplotypes (Table 5). The differences were observed in the control region, RNR1, TV, TL1, ND1, TA, TY, ATP6, ND4, ND5, ND6, and CYB. The greatest number of different regions was observed between P01-H and P08-H, where five different regions were observed. The largest number of pairwise different variants was also observed in the same comparison pair, where five different PHPs and two different LHPs were observed (Figure 7).

Inherited PHPs were observed in Family1 (152Y), Family8 (3386Y), P08-P15 (9083Y), and P12-P18 (14215Y); conversely, de novo and disappearing PHPs were the majority (Table 3). The pairwise MAF differences between the mother and offspring were 10.19% and 12.54% in 3386Y and 9083Y, while the values were 1.31% in 152Y and 0.51% in 14215Y (Table 3).

**Figure 8:**
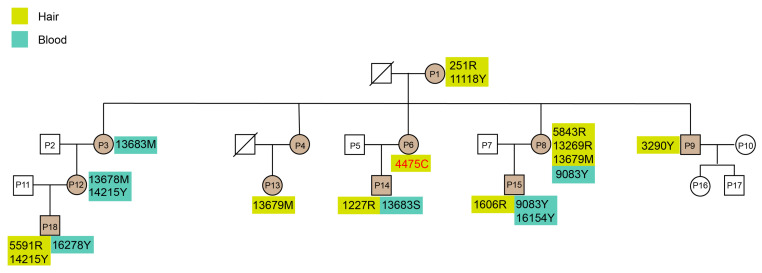
Point heteroplasmy variants in the four-generation pedigree. Variants with a yellow background were observed in hair shafts, and variants with a blue background were observed in blood samples. Individuals with a white background were not included in this study.

**Figure 9:**
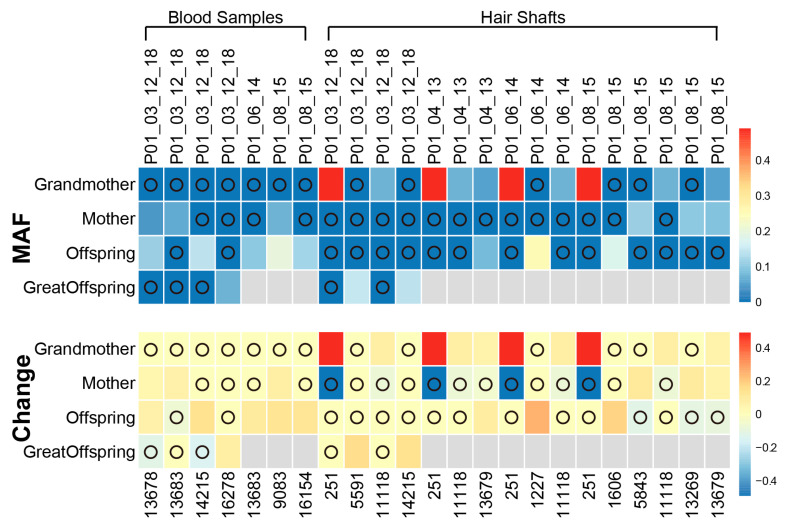
PHP and MAF changes during transmission. The top panel shows the MAF of the PHP for each sample. The bottom panel shows the MAF changes during transmission. A circle indicates that the heteroplasmy disappeared in the sample.

A total of six groups of immediate three-generational family members (i.e., grandmother–mother–offspring) and one group of immediate four-generational family members (i.e., grandmother–mother–offspring–great-grandson) were included in this study. PHPs were observed in both the blood samples and hair shafts in the four-generational group and two three-generational groups and were also observed in the hair shafts of one three-generational group (Figure 8 and Figure 9). The transmission of PHPs 13678M and 9083Y was observed in the blood samples of the grandmother–mother–offspring–great-offspring group (P01–P03–P12–P18) and a grandmother–mother–offspring group (P01–P08–P15), respectively. Additionally, the transmission of PHP 13679M was observed in the hair shafts of the P01–P08–P15 group (Figure 9). The MAF changes during transmission among the generations are shown in Figure 9. As shown in this figure, de novo/disappearing PHPs made up the majority of the PHPs, and no PHP was transmitted throughout the pedigree.

### 3.5. Variant Differences between Maternal Relatives in Three/Four-Generation Pedigrees

**Figure 10:**
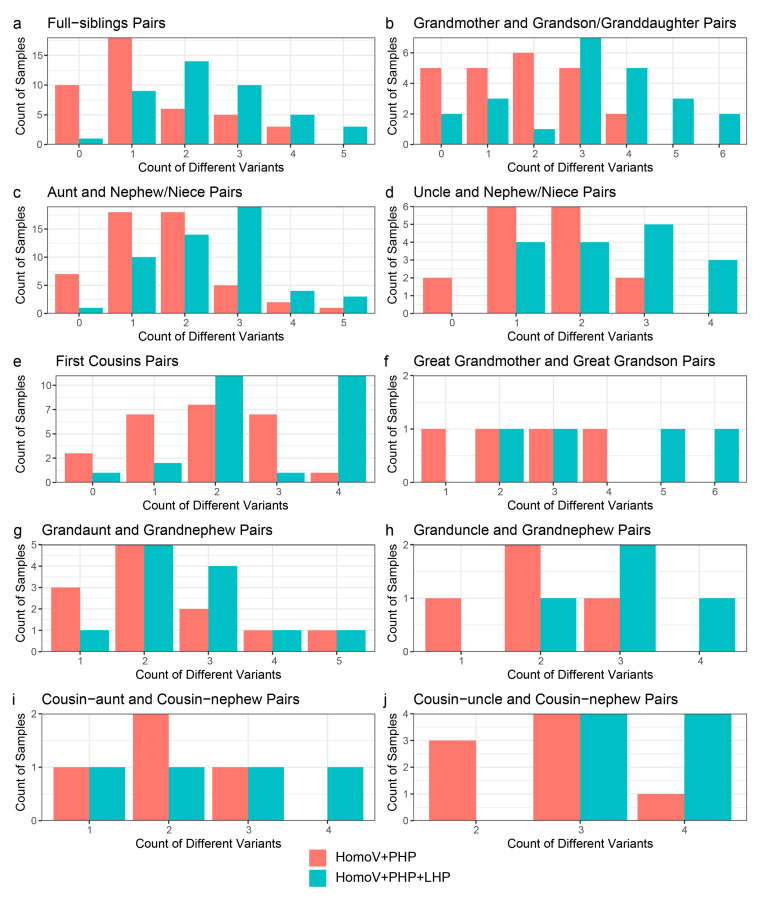
Counts of different variants in the ten types of maternal relationships.

Besides the mother–offspring pairs, comparisons were also carried out in 10 other types of maternal relationships (Table 6). Similar to the mother–offspring pairs, when only homoplasmic variants were compared, an mtGenome haplotype difference was observed only in pairs where P06-H was included (eight in full-sibling pairs, six in maternal aunt and maternal nephew/niece pairs, and two in maternal grandaunt and maternal grandnephew pairs). When taking the heteroplasmic variants into consideration, different mtDNA haplotypes were observed in most of the comparisons, especially in the comparisons of third-degree relationships, where differences were observed in almost all comparisons (Table 6). The involved regions were the same as those in the mother–offspring pairs. The number of different comparisons for each type of maternal relative is shown in Figure 10 and Supplementary Figure S5.

## 4. Discussion

### 4.1. Sequencing Overview

In this study, we used a fire-new mtDNA Whole Genome Kit that was designed with small amplicons (averaging at 131 bp) [40,42] to sequence the mtGenome in blood samples and hair shafts. Amplification of small amplicons is more effective for degraded samples, while the risk of NUMT contamination also increases [38,39]. The two-PCR approach with tiled amplicons can facilitate the confirmation of variants that reside at the primer-binding sites: when a primer-binding site mutation exists under a primer in one primer set, that variant can be reliably detected in amplicons extended from the companion primer set [42]. This strategy ensures the successful detection of the mtGenome with a high genome coverage. However, in our study, we still observed a low read depth or even no base call at some nucleotide fragments in most of the samples, and these fragments overlapped with the validation study of the ForenSeq mtGenome Kit [42]. This phenomenon may be caused by the failure of amplification at these positions. 

In this study, the quantity of the input DNA and the processes before library pooling followed the manufacturer’s instructions completely. When using the recommended number of pooling libraries (16 libraries) and the recommended volume of pooled libraries (5 μL) that was added to the Miseq sequencing reagent cartridge, 73.33% of the sequenced libraries (11/15, negative control not included) showed a total read depth of lower than 300,000×. To maximize the data coverage and quality, we lowered the number of pooling libraries to 12 and increased the volume of pooled libraries to 5.3 μL in the later sequencing runs. These adjustments proved to be effective at increasing the total read depth and mtGenome coverage throughout the results of run 1, run 4, and run 5 (Figure 1). Thus, reducing the number of pooling libraries and increasing the volume of pooled libraries can help to obtain more sequencing reads and a larger coverage, which is meaningful for challenging samples. On the other hand, the total read depths of the anticoagulant blood samples (runs 4 and 5, collected using an EDTA anticoagulant tube) were only half as deep as those for the blood stains (run 3, collected using a sterilized filter) and hair shafts (run 2). Excluding the difference in the concentration of the input DNA and normalized libraries, this phenomenon may be caused by the in vitro time of the samples and the storage methods, as the anticoagulant blood samples were in vitro for three years and the freezing–thawing process can decrease the mitochondrial membrane potential and lead to damage to the mitochondria [51]. This phenomenon reminded us to store the material under relatively stable conditions and avoid repeated freezing and thawing, and we recommend that the materials be prepared as dry stains and stored in dry and constant temperature conditions if possible.

This kit performed well in terms of the sequence strand balance. The percentage of positions with strand bias (1.68%) was lower than that in samples sequenced using the Ion Torrent PGM platform (10%) [52], the Ion Torrent S5 platform (16%) [53], and MiSeq FGx (3.06%, with 2 long mtDNA amplicons) [1]. Due to the poly-C stretches, the forward sequences between nps 303 and 347 did not meet the alignment requirements and were soft-clipped, thus leading to sequencing reads with only a reverse direction [42].

Completely consistent homoplasmic variants were observed in the HL60 samples in our study, previous studies [42,54], and the SRM2392-I certificate [55], while for three nucleotide positions (nps 2445, 4821, and 12,071), the involved PHPs were different (Supplementary Table S3). At np 2445, the genotype was T2445 in the SRM2392-Icertificate and our study, while it was 2445Y in [42,54]. At np 4821, the other 3 studies reported an rCRS genotype (G4821), while our study showed PHP 4821R (7.4% of allele A). At np 12,071, the other 3 studies reported PHP 12071Y, while in our study, an rCRS genotype (T12071) was observed, with a frequency of 1.1% for allele C, and was not identified as a PHP allele. According to the study by Cihlar et al. [54], lot-to-lot variation in control DNAs was observed; thus, it is not surprising that different PHPs were observed in the HL60 samples that were from different lots. Additionally, with the high sensitivity and resolution power of MPS, we were able to distinguish more PHPs with lower thresholds. In the study herein and a validation study of the same mtGenome detection kit [42], 6% was used for the PHP calling, while the value was 10% in [54], and the haplotypes in the SRM2392-I certificate were confirmed using Sanger-type sequencing. Moreover, the sequencing platform, sequencing chemistry [35,56], and analysis software [57] can also cause variability. In spite of the differences in the nucleotide positions of the PHPs, the sequencing results of HL60 in this study are reliable.

Due to the high sensitivity of the mtGenome multiplex and the unavoidable inclusion of aerosol during the pipetting process, sporadic base calls were unavoidable in the negative controls. When the default variant calling thresholds were used, no variant call was observed in NC-EXB and NC-AMP, which indicates ignorable contamination during the DNA extraction process of the blood samples and the library construction and sequencing process. On the contrary, remarkable variants were present in the negative control of the hair shaft extraction (NC-EXH). Among the 22 observed variants, 8 overlapped with HL60 and P01-H. When compared with all the samples in this study, there were still nine variants that were not traced. Additionally, these variants were not recorded in the EMPOP database [41], and many unexpected variants were observed when assigning the haplogroup of NC-EXH. Thus, we inferred that the contamination of NC-EXH was neither single-sourced nor researched-sample-sourced. According to the consistent haplotypes of the blood samples and hair shafts from the same individual and the consistent haplotypes between maternal relatives (heteroplasmic variants were not considered), we can conclude that the source of contamination had little influence on the detected samples in this study and can be ignored when performing variant calls in hair shaft samples. Overall, the ForenSeq mtGenome Kit is suitable for mtDNA detection in blood samples and challenging samples such as rootless hair shafts, and the sequencing results are reliable in this study.

### 4.2. mtDNA Polymorphism and Heteroplasmy

The distribution of variants in the mtGenome in this study was similar to that in the North Han Chinese population reported by Zhou et al. [9] and in the Shanghai Han Chinese population reported by Ma et al. [52]. Similar distributions of variants in the control region and coding region were observed in previous studies (72.16% ± 1.10% on average) [9,10,23,52]. In this study, 100% unique haplotypes were observed in the 10 pedigrees when considering the control region, which suggested the high polymorphism of the control region, even if only 1122 bps of the mtDNA sequence were included (Figure 6). Meanwhile, some studies also reported an increase in the power of evidence when comparing the mtGenome with the control region, wherein the haplotype diversity increased by 0.02% to 0.21% and the random match probability decreased by 2.02% to 35% [9,10,11]. In this study, relatively high polymorphisms were observed in the coding regions of ATP6, ND5, CYB, ND2, and CO1, where there were nine, nine, eight, seven, and seven types of haplotypes, respectively (Figure 6). This suggests that we can selectively detect the control region and these high-polymorphism coding regions to balance the discrimination power and cost.

However, no overlapping PHPs have been observed in previous studies [9,10,23,52], which indicates a random happening of the nucleotide positions of PHPs [9,58,59]. Moreover, different from Li et al.’s observation that most PHPs (MAF = 2%) were distributed in the control region [17] in 12 types of tissues from 152 individuals, 83.33% of the PHPs were observed in the coding region in this study, and this is in agreement with previous studies using MPS platforms (84.38% in [52], 75.00% in [10], 70.83% in [9], and 64.49% in [23]). No shared PHPs were observed in the blood and hair shaft pairs as only 11 individuals were detected in this study. The haplogroup assignments were consistent between the blood samples and corresponding hair shafts and were also consistent among relatives in the same pedigree, indicating that the variants at the heteroplasmic positions are not the expected variants for haplogroup assignment and will not influence the assignment results. 

Extreme strand bias was only observed at nps 303–315 and np 16193.1 in the 16,180–16,194 fragment. 310T interrupted the C-stretches at nps 303–315, which resulted in nine consecutive Cs in this region. Variant 309.1c was called when both the read depth and frequency of reads with the insertion of C and reads without the insertion of C (reference reads) reached the thresholds; otherwise, variant 309.1C was called. A relatively high frequency occurred when the total read depth was low, even with a read count that was less than 45 reads (e.g., 20×/100× = 20%); nonetheless, a homoplasmic variant was called. A similar situation existed in the np 955–966 and np 16,180–16,194 fragments. An N-1 stutter in np 16,189 was observed, and the count of stutter reads was 13.74% of that of the parental allele. This is similar to the mechanism that results in stutter products associated with the detection of short tandem repeat (STR) markers [60]. Although the dinucleotide repeat (AC)5 was observed in the 514–524 fragment, an average frequency of 84.52% ± 0.98% of (AC)4 was also observed. The (AC)4 repeat is more frequent in Asian populations [61]. The 9 bp deletion between nps 8272 and 8280 that has been deeply investigated in the Chinese population [62] was also reported in this study. Positions 12,418–12,425 are an 8 bp polyadenine stretch, and a mixture of molecules in this region has been previously described in a report on mtDNA heteroplasmy from MPS data [22]. Just et al. also reported that 88.8% of 588 samples had detectible LHP around position 12,425 based on Sanger-type sequencing [11].

Though the sequencing-by-synthesis mechanism of the Illumina sequencing platform can reduce the risk of sequencing error in the polymeric region, the lower thresholds of variant calls (e.g., 6% in UAS v2.1) may increase the complexity of variants in regions with length variations. Thus, we recommend that caution be taken when classifying LHP calls and that the minimum variant frequency of length variation should be raised (e.g., to 20% for insertions and 30 % for deletions [23,29]). Additionally, we suggest that the manufacturer provides an independent option for the threshold settings of length variant calls in the UAS updates.

### 4.3. Variants in Maternal Relatives

The variant transmission of the mtGenome between the maternal family members in this study still followed maternal heredity. Meanwhile, we also observed differences that were mainly caused by heteroplasmic variants. These variants are de novo or disappearing variants in the lineage. The transmission of inherited heteroplasmic variants can improve the weight of evidence when evaluating individuals from the same maternal lineage, while different heteroplasmies may have the potential to distinguish individuals from the same maternal ancestry, especially when the heteroplasmic variants are only shared in tissues of the particular individual [24].

In this study, the six groups of immediate three-generational family members and the group of immediate four-generational family members were from only two maternal lineages; thus, limited information on the characteristics of PHP transmission was found. PHP that was transmitted from the grandmother to the third- or fourth-generation member was not observed. Meanwhile, in the observed inherited PHPs, the direction and magnitude of the frequency change during transmission were moderate and seemed to be random, and this is consistent with Liu et al.’s research on sixteen four-generation pedigrees [33]. Zaidi et al. demonstrated that divergence between the mother and offspring increases with the mother’s age at childbirth [32], while in this study, divergence was not observed among the mother and her five offspring, where the first offspring was ten years older than the fifth offspring. Heteroplasmy allele frequencies can be affected by the germline bottleneck, by the potential decrease in the content of mtDNA during embryonic development, and by selection [32,63]. No correlation between the MAF changes among the transmissions was observed, suggesting that most heteroplasmic variants were functionally neutral or mildly deleterious and were not eliminated by selection [33]. 

In this study, though only four inherited PHPs were observed, the length variations were inherited in most of the maternal relatives, which can also improve the matching probability of the same maternal origin among the relatives. Optimizing the thresholds for LHP variant calling or using a major molecule comparison helps to decrease the complexity of a variant mixture and can support the same maternal origin relationship further. Meanwhile, more pedigree studies are needed to further confirm the observation that more variant differences existed between the relatives of third-degree relationships. 

As for the mtGenome haplotype comparisons between maternal relatives, the revised guidelines by the ISFG recommends that differences in both PHPs and LHPs do not constitute evidence for excluding two identical haplotypes deriving from the same source or the same maternal lineage [47]. However, Connell et al. proved that the sequence comparison guidelines for the mtDNA control region recommended by SWGDAM [8] are also suitable for multigeneration whole mtGenome analysis, wherein samples differing at two or more nucleotide positions (excluding length heteroplasmy) can be excluded when coming from the same maternal lineage (reported as ‘exclusion’), samples differing at a single position should only be reported as ‘inconclusive’, and samples having the same sequence should be reported as ‘cannot exclude’. They also recommended that caution should be taken when classifying heteroplasmic changes as differences for human identification [64]. As the ForenSeq mtGenome Kit is highly sensitive and has the potential to distinguish LHPs, we wondered how many differences between maternal relatives would be observed using this panel. Thus, we implemented three levels of comparison, wherein the first comparison considered homoplasmic variants only, the second comparison considered both homoplasmic variants and point heteroplasmy, and the third comparison considered both homoplasmic variants and heteroplasmies (both PHPs and LHPs). In this study, following the guidelines, when comparing the haplotypes with homoplasmic variants only, 100% of the reports obtained were ‘cannot exclude’ in the grandmother and grandson/granddaughter pairs, uncle and nephew/niece pairs, first-cousin pairs, great-grandmother and great-grandson pairs, granduncle and grandnephew pairs, cousin-aunt and cousin-nephew pairs, and cousin-uncle and cousin-nephew pairs. A report of ‘inconclusive’ was obtained in pairs of the P06-H sample, with four pairs in mother and offspring pairs, eight pairs in full-sibling pairs, six pairs in aunt and nephew/niece pairs, and two pairs in grandaunt and grandnephew pairs. No report of ‘exclusion’ was obtained under this comparison condition. When comparing the haplotypes with both homoplasmic variants and PHPs, a 33.33% to 100% count of ‘exclusion’ was reported in the 11 types of maternal relationships. Furthermore, when LHPs were also compared, the percentage of ‘exclusion’ increased from 62.26% to 100% in the 11 types of maternal relationships. Especially in the great-grandmother and great-grandson pairs, granduncle and grandnephew pairs, and cousin-uncle and cousin-nephew pairs, 100% ‘exclusion’ was reported (Supplementary Table S4). Similarly, in the study by Connell et al., wherein mtGenome haplotypes of 2339 maternal pairs from 18 meioses were compared, the prevalence of inconclusive identification increased by 6%, and the prevalence of false exclusions was 0.34% when PHPs were considered [64]. Since they used a higher MAF (10%) than ours, fewer PHPs were observed (6.67%), resulting in fewer mtDNA differences than in our study when PHPs were considered. Overall, in this study, the comparison guidelines were suitable for the mtGenome haplotype comparison between all maternal relatives and throughout the multigenerational comparisons when only homoplasmic variants were considered. Given the high false exclusion ratio, we suggest that different heteroplasmic variants should not be counted as inconsistencies when performing mtDNA haplotype comparisons, while consistent heteroplasmic variants can be considered as enhanced evidence for maternal lineage confirmation. Additionally, this is consistent with the recommendations of the ISFG [47].

## 5. Conclusions

In recent years, some mtDNA detection panels have been established for forensic use based on MPS platforms. In this study, we used a new mtGenome detection panel, the ForenSeq mtGenome Kit, to detect blood samples and hair shafts from maternal-pedigree individuals. Firstly, we demonstrated the effectiveness of this panel in blood and hair shaft detection through the deep read depth and complete mtGenome coverage. Secondly, the ForenSeq mtGenome Kit plus ForenSeq UAS v2.1 software can distinguish between PHPs and LHPs clearly, while the threshold setting function for LHP needs to be improved. Thirdly, we observed a stable transmission of homoplasmic variants among maternal pedigrees and variable differences in heteroplasmic variants between maternal relatives. Random MAF changes among three/four-generation pedigrees were also observed. Lastly, we proved that a high risk of false exclusion will present itself if the differences in both PHPs and LHPs are included for exclusion. In the future, hair shafts from different body parts and various types of tissues, especially challenge materials, should be investigated to obtain more comprehensive knowledge of the mtGenome. 

## Figures and Tables

**Table 1:** Loaded libraries and sequence quality metrics of five sequencing runs.

Run	Number of Pooled Libraries	Volume of Loaded Libraries	Sample Type	Cluster Density (k/mm^2^)	Cluster Passing Filter	Phasing	Pre-Phasing
Run 1	14 + 2 (HL-60, NC-AMP)	5 μL	Blood	1163	92.98%	0.148%	0.057%
Run 2	11 + 1 (NC-EXH)	5.3 μL	Hair shaft	1762	87.87%	0.143%	0.054%
Run 3	11 + 1 (NC-EXB)	5.3 μL	Blood	1798	86.18%	0.154%	0.063%
Run 4	12	5.3 μL	Blood	1491	89.19%	0.134%	0.039%
Run 5	12	5.5 μL	Replicates	1589	88.59%	0.152%	0.045%

**Table 2:** Average read depth, count of variants, and count of variant types in control region and the 37 gene-coding regions.

Gene ^a^	Region (nps ^b^)	Average Depth (*n* = 60)	Count of Variants (*n* = 33)	Types of Variant/Heteroplasmy (*n* = 33)
CR	16,024–576	7686.52 ± 8739.42	396	56/11
RNR1	648–1601	5311.63 ± 4254.04	136	12/4
TV	1602–1670	1999.34 ± 399.66	0	0
RNR2	1671–3229	4187.71 ± 4295.22	54	5
TL1	3230–3304	4844.61 ± 1523.7	0	0
ND1	3307–4262	1980.16 ± 1324.67	25	7/1
TI	4263–4331	1120.59 ± 631.68	0	0
TQ	4329–4400	975.27 ± 766.07	0	0
TM	4402–4469	2440.76 ± 849.32	0	0
ND2	4470–5511	6883.6 ± 6851.97	90	13
TW	5512–5579	2125.61 ± 1110.14	0	0
TA	5587–5655	1675.44 ± 1871.54	2	1
TN	5657–5729	6784.35 ± 2954.64	0	0
TC	5761–5826	8920.13 ± 4975.43	2	1
TY	5826–5891	4188.07 ± 2766.39	0	0
CO1	5904–7445	4511.09 ± 3434.52	61	10
TS1	7446–7514	1705.74 ± 432.68	0	0
TD	7518–7585	751.32 ± 242.26	0	0
CO2	7586–8269	12,357.99 ± 9801.41	21	5
TK	8295–8364	313.25 ± 27.54	0	0
ATP8	8366–8572	1895.9 ± 1345.62	19	3
ATP6	8527–9207	6179.5 ± 6712.78	82	10/1
CO3	9207–9990	4442.88 ± 3478.39	26	5
TG	9991–10,058	2334.23 ± 1436.11	2	1
ND3	10,059–10,404	4387.68 ± 3476.78	36	5
TR	10,405–10,469	2652.93 ± 560.33	0	0
ND4L	10,470–10,766	8878.17 ± 5405.51	6	3
ND4	10,760–12,137	5635.59 ± 6647.66	62	5
TH	12,138–12,206	2247.28 ± 2864.1	0	0
TS2	12,207–12,265	7589.28 ± 91.77	0	0
TL2	12,266–12,336	5553.71 ± 6516.03	0	0
ND5	12,337–14,148	7048.7 ± 8283.93	78	20/4
ND6	14,149–14,673	2510.71 ± 1729.39	15	4/1
TE	14,674–14,742	1431.75 ± 261.85	0	0
CYB	14,747–15,887	9228.57 ± 15,169.37	132	11/1
TT	15,888–15,953	4776.47 ± 2259.4	0	0
TP	15,956–16,023	21,029.81 ± 2391.2	0	0

^a^ CR: the control region of mitochondrial DNA. TF: mitochondrially encoded tRNA phenylalanine. RNR1: mitochondrially encoded 12S RNA. TV: mitochondrially encoded tRNA valine. RNR2: mitochondrially encoded 16S RNA. TL1: mitochondrially encoded tRNA leucine 1 (UUA/G). ND1: mitochondrially encoded NADH dehydrogenase 1. TI: mitochondrially encoded tRNA isoleucine. TQ: mitochondrially encoded tRNA glutamine. TM: mitochondrially encoded tRNA methionine. ND2: mitochondrially encoded NADH dehydrogenase 2. TW: mitochondrially encoded tRNA tryptophan. TA: mitochondrially encoded tRNA alanine. TN: mitochondrially encoded tRNA asparagine. TC: mitochondrially encoded tRNA cysteine. TY: mitochondrially encoded tRNA tyrosine. CO1: mitochondrially encoded cytochrome c oxidase I. TS1: mitochondrially encoded tRNA serine 1 (UCN). TD: mitochondrially encoded tRNA aspartic acid. CO2: mitochondrially encoded cytochrome c oxidase II. TK: mitochondrially encoded tRNA lysine. ATP8: mitochondrially encoded ATP synthase 8. ATP6: mitochondrially encoded ATP synthase 6. CO3: mitochondrially encoded cytochrome c oxidase III. TG: mitochondrially encoded tRNA glycine. ND3: mitochondrially encoded NADH dehydrogenase 3. TR: mitochondrially encoded tRNA arginine. ND4L: mitochondrially encoded NADH 4L dehydrogenase. ND4: mitochondrially encoded NADH dehydrogenase 4. TH: mitochondrially encoded tRNA histidine. TS2: mitochondrially encoded tRNA serine 2 (AGU/C). TL2: mitochondrially encoded tRNA leucine 2 (CUN). ND5: mitochondrially encoded NADH dehydrogenase 5. ND6: mitochondrially encoded NADH dehydrogenase 6. TE: mitochondrially encoded tRNA glutamic acid. CYB: mitochondrially encoded cytochrome b. TT: mitochondrially encoded tRNA threonine. TP: mitochondrially encoded tRNA proline. ^b^ nps: nucleotide positions.

**Table 3:** Point heteroplasmies observed in this study.

Position	Individual	Type	Reference Allele	Reference Frequency	Variant Allele	Variant Frequency	Genotype	MAF	Region	Type of PHP
152	Family1-M	Blood	T	29.76%	C	70.24%	152Y	29.76%	CR	Inherited
152	Family-C	Blood	T	31.07%	C	68.93%	152Y	31.07%	CR	Inherited
251	P01-H	Hair	G	50.91%	A	49.09%	251R	49.09%	CR	Disappearing
1227	P14-H	Hair	G	75.31%	A	24.69%	1227R	24.69%	RNR1	De novo
1606	P15-H	Hair	G	81.84%	A	18.16%	1606R	18.16%	TV	De novo
3290	P09-H	Hair	T	84.79%	C	15.21%	3290Y	15.21%	TL1	De novo/disappearing
3386	Family8-M	Blood	T	64.05%	C	35.95%	3386Y	35.95%	ND1	Inherited
3386	Family8-C	Blood	T	74.24%	C	25.76%	3386Y	25.76%	ND1	Inherited
4776	P09-H	Hair	G	85.36%	C	14.64%	4776S	14.64%	ND2	De novo/disappearing
5591	P18-H	Hair	G	85.00%	A	15.00%	5591R	15.00%	TA	De novo
5843	P08-H	Hair	A	89.16%	G	10.84%	5843R	10.84%	TY	De novo/disappearing
9083	P08-B	Blood	T	92.69%	C	7.30%	9083Y	7.30%	ATP6	Inherited
9083	P15-B	Blood	T	80.16%	C	19.84%	9083Y	19.84%	ATP6	Inherited
11,118	P01-H	Hair	T	93.03%	C	6.97%	11118Y	6.97%	ND4	Disappearing
12,361	Family3-M	Blood	A	32.08%	G	67.92%	12361R	32.08%	ND5	Disappearing
13,269	P08-H	Hair	A	90.48%	G	9.52%	13269R	9.52%	ND5	De novo/disappearing
13,678	P12-B	Blood	C	89.29%	A	10.71%	13678M	10.71%	ND5	De novo
13,679	P08-H	Hair	C	90.66%	A	9.34%	13679M	9.34%	ND5	De novo/disappearing
13,679	P13-H	Hair	C	92.13%	A	7.87%	13679M	7.87%	ND5	De novo
13,683	P03-B	Blood	C	93.61%	A	6.39%	13683M	6.39%	ND5	De novo/disappearing
13,683	P14-B	Blood	C	89.75%	G	10.25%	13683S	10.25%	ND5	De novo
14,215	P12-B	Blood	T	85.91%	C	14.09%	14215Y	14.09%	ND6	Inherited
14,215	P18-H	Hair	T	86.42%	C	13.58%	14215Y	13.58%	ND6	Inherited
15,115	Family7-M	Blood	T	91.28%	C	8.72%	15115Y	8.72%	CYB	Disappearing
16,154	P15-B	Blood	T	88.41%	C	11.59%	16154Y	11.59%	CR	De novo
16,278	P18-B	Blood	C	92.94%	T	7.06%	16278Y	7.06%	CR	De novo

**Table 4:** Variants that caused the length variations in the mtDNA haplotypes.

Position	Variant Type	Haplotype Reported with UAS	Count of Samples	LHP
302–315 ^a^	A-7C-T-6C-G	315.1C	8	NO
302–315	A-8C-T-6C-G	309.1C, 315.1C	21	NO
302–315	A-8C-T-6C-G	309.1c, 315.1C	12	YES
302–315	A-9C-T-6C-G	309.1C, 309.2c, 315.1C	2	YES
955–966 ^b^	A-12C-A	961C, 965.1C, 965.2c	2	YES
955–966	A-12C-A	961C, 965.1c, 965.2c	15	YES
955–966	A-12C-A	961c, 965.1c, 965.2c	3	YES
955–966	A-13C-A	961C, 965.1c, 965.2c, 965.3c	4	YES
12,417–12,426 ^c^	C-9A-C	12425.1a	1	YES
16,180–16,194 ^d^	2A-13C-A	16182C, 16183C, 16189C, 16193.1c	2	YES
16,180–16,194	3A-12C-A	16183C, 16189C, 16193.1c	2	YES
249	249DEL	249DEL	4	NO
514–524 ^e^	C (AC)5	523a524c	30	YES
8272–8280 ^f^	8272–8274DEL8275c8276c8277t8278c8279t8280a		2	YES

^a^ The type of rCRS at nps 302–315 is ‘A-7C-T-5C-G’, and the cause of length variation is base insertion; ^b^ the type of rCRS at nps 955–966 is ‘A-5C-T-4C-A’, and the cause of length variation is base insertion; ^c^ the type of rCRS at nps 12,417–12,426 is ‘C-8A-C’, and the cause of length variation is base insertion; ^d^ the type of rCRS at nps 16,180–16,194 is ‘4A-5C-T-4C-A’, and the cause of length variation is base insertion; ^e^ the type of rCRS at nps 514–524 is ‘C (AC)5′, and the cause of length variation is base deletion; ^f^ the type of rCRS at nps 8272–8280 is ‘CCCCCTCTA’, and the cause of length variation is base deletion.

**Table 5:** Different regions and different heteroplasmic variants between the mtDNA haplotypes of mother and offspring pairs.

Mother	Offspring	Sample Type	Count of Consistent Region	Different Region	Count of Different Heteroplasmic Variants (PHP/LHP) ^a^	Details of Different Heteroplasmic Variants (Mother/Offspring) ^b^
Family1-M	Family1-C	B-B ^c^	38		0	
Family2-M	Family2-C	B-B	38		0	
Family3-M	Family3-C	B-B	36	ND5; CR	1/1	12361R/-; 309.1c/309.1C
Family4-M	Family4-C	B-B	37	RNR1	0/1	961c/-
Family5-M	Family5-C	B-B	38		0	
Family6-M	Family6-C	B-B	38		0	
Family7-M	Family7-C	B-B	38		0	
Family8-M	Family8-C	B-B	38		0	
JX-N	JX-M1	B-B	38		0	
JX-N	JX-M2	B-B	37	CR	0/1	309.1c/309.1C
JX-M1	JX-C1	B-B	38		0	
JX-M1	JX-C2	B-B	38		0	
JX-M2	JX-C3	B-B	37	CR	0	
P01-B	P03-B	B-B	36	ND5; RNR1	1/1	-/13683M; 961c/961C; -/965.3c
P01-B	P04-B	B-B	38		0	
P01-B	P06-B	B-B	37	RNR1	0/1	961c/961C; -/965.3c
P01-B	P08-B	B-B	36	ATP6; RNR1	1/1	-/9083Y; 961c/961C
P01-B	P09-B	B-B	36	CR; RNR1	0/2	309.1C/309.1c; 961c/961C
P03-B	P12-B	B-B	35	ND5; ND5; ND6; RNR1	3/1	13683M/-; -/13678M; -/14215Y; 965.3c/-
P04-B	P13-B	B-B	37	RNR1	0/1	961c/961C
P06-B	P14-B	B-B	37	ND5	1/0	-/13683S
P08-B	P15-B	B-B	37	CR	1/0	-/16154Y
P12-B	P18-B	B-B	35	ND5; ND6; CR	3/0	-/13678M; -/14215Y; -/16278Y
P01-B	P03-H	B-H ^d^	36	CR; RNR1	0/2	309.1C/309.1c; -/965.3c
P01-B	P04-H	B-H	37	RNR1	0/1	961c/961C
P01-B	P06-H	B-H	36	RNR1	0/1	961c/961C
P01-B	P08-H	B-H	35	TY; ND5; ND5; RNR1	3/1	-/5843R; -/13269R; -/13679M; 961c/961C
P01-B	P09-H	B-H	35	TL1; CR; RNR1	1/2	-/3290Y; 309.1C/309.1c; 961c/961C
P03-B	P12-H	B-H	36	ND5; RNR1	1/1	13683M/-; 965.3c/-
P04-B	P13-H	B-H	35	ND5; CR; RNR1	1/2	-/13679M; 309.1C/309.1c; 961c/961C
P06-B	P14-H	B-H	36	RNR1; CR; RNR1	1/2	-/1227R; 309.1C/309.1c; 965.3c/-
P08-B	P15-H	B-H	36	ATP6; TV	2/1	9083Y/-; -/1606R; 965.1c 965.2c/- -
P12-B	P18-H	B-H	36	ND5; TA	2/0	13678M/-; -/5591R
P01-H	P03-H	H-H ^e^	35	CR; ND4; CR; RNR1	2/2	251R/-; 11118Y/-; -/309.1c; -/965.3C
P01-H	P04-H	H-H	36	CR; ND4; CR	2/1	251R/-; 11118Y/-; -/309.1C
P01-H	P06-H	H-H	35	CR; ND4; CR	2/1	251R/-; 11118Y/-; -/309.1C
P01-H	P08-H	H-H	33	CR; ND4; TY; ND5; ND5; CR; RNR1	5/2	251R/-; 11118Y/-; -/5843R; -/13269R; -/13679M; -/309.1C; 965.1C/965.1c
P01-H	P09-H	H-H	34	CR; ND4; TL1; CR; RNR1	3/2	251R/-; 11118Y/-; -/3290Y; -/309.1c; 965.1C/965.1c
P03-H	P12-H	H-H	36	CR; RNR1	0/2	309.1c/309.1C; 965.3c/-
P04-H	P13-H	H-H	36	ND5; CR	1/1	-/13679M; 309.1C/309.1c
P06-H	P14-H	H-H	35	RNR1; CR	1/1	-/1227R; 309.1C/309.1c
P08-H	P15-H	H-H	34	TY; ND5; ND5; TV; RNR1	4/1	5843R/-; 13269R/-; 13679M/-; -/1606R; 965.1c 965.2c/- -
P12-H	P18-H	H-H	36	TA; ND6	2/0	-/5591R; -/14215Y
P01-H	P03-B	H-B ^f^	34	CR; ND4; ND5; CR; RNR1; RNR1	3/2	251R/-; 11118Y/-; -/13683M; -/309.1C; 965.1C/965.1c; -/965.3c
P01-H	P04-B	H-B	35	CR; ND4; CR; RNR1	2/2	251R/-; 11118Y/-; -/309.1C; 961C/961c; 965.1C/965.1c
P01-H	P06-B	H-B	34	CR; ND4; CR; RNR1	2/2	251R/-; 11118Y/-; -/309.1C; 961C/961c; 965.1C/965.1c; -/965.3c
P01-H	P08-B	H-B	34	CR; ND4; ATP6; CR; RNR1	3/2	251R/-; 11118Y/-; -/9083Y; -/309.1C; 965.1C/965.1c
P01-H	P09-B	H-B	35	CR; ND4; CR; RNR1	2/2	251R/-; 11118Y/-; -/309.1c; 965.1C/965.1c
P03-H	P12-B	H-B	34	ND5; ND6; CR; RNR1	2/2	-/13678M; -/14215Y; 309.1c/309.1C; 965.3c/-
P04-H	P13-B	H-B	38		0	
P06-H	P14-B	H-B	35	ND5; RNR1	1/1	-/13683S; -/965.3c
P08-H	P15-B	H-B	34	TY; ND5; ND5; ATP6; CR	5/0	5843R/-; 13269R/-; 13679M/-; -/9083Y; -/16154Y
P12-H	P18-B	H-B	37	CR	1/0	-/16278Y

^a^ The value in front of the slash is the count of different PHPs, and the value behind the slash is the count of different LHPs; ^b^ the variants in front of the slash are from the mother, and the variants behind the slash are from the offspring; ^c^ ‘B-B’ represents the mother’s blood sample and the offspring’s blood sample pair; ^d^ ‘B-H’ represents the mother’s blood sample and the offspring’s hair shaft pair; ^e^ ‘H-H’ represents the mother’s hair shaft and the offspring’s hair shaft pair; ^f^ ‘H-B’ represents the mother’s hair shaft and the offspring’s blood sample pair.

**Table 6:** Different mtGenome haplotypes in ten types of maternal relationships.

Degree of Kinship	Type of Maternal Relationship	Count of Pairs	Count of Comparisons	Count of Different Comparisons (HomoV) ^a^	Count of Different Comparisons (HomoV + PHP) ^b^	Count of Different Comparisons (HomoV + PHP + LHP) ^c^
1st	Full-siblings	11	42	8 (19.05%)	32 (76.19%)	41 (97.62%)
2nd	Maternal grandmother and maternal grandson/granddaughter	8	23	0 (0%)	18 (78.26%)	21 (91.30%)
2nd	Maternal aunt and maternal nephew/niece	15	51	6 (11.76%)	44 (86.27%)	50 (98.04%)
2nd	Maternal uncle and maternal nephew/niece	4	16	0 (0%)	14 (87.50%)	16 (100%)
3rd	Maternal first cousins	8	26	0 (0%)	23 (88.46%)	25 (96.15%)
3rd	Maternal great grandmother and maternal great grandson	1	4	0 (0%)	4 (100%)	4 (100%)
3rd	Maternal grandaunt and maternal grandnephew	3	12	2 (16.67%)	12 (100%)	12 (100%)
3rd	Maternal granduncle and maternal grandnephew	1	4	0 (0%)	4 (100%)	4 (100%)
4th	Maternal cousin-aunt and maternal cousin-nephew	1	4	0 (0%)	4 (100%)	4 (100%)
4th	Maternal cousin-uncle and maternal cousin-nephew	2	8	0 (0%)	8 (100%)	8 (100%)

^a^ Only homoplasmic variants were compared; ^b^ both homoplasmic variants and PHPs were compared; ^c^ homoplasmic variants, PHPs, and LHPs were compared.

## Data Availability

The data presented in this study are available on request from the corresponding author. The data are not publicly available due to the restrictions of privacy and the law.

## Supplementary Materials

The following supporting information can be downloaded at: https://www.mdpi.com/article/10.3390/genes14040912/s1, Figure S1: Family tree of the four-generation and three-generation pedigree; Figure S2: Relative read depth of three negative controls and HL60; Figure S3: Total read depth and mtGenome coverage of the twice-sequenced libraries; Figure S4: Distribution of PHP count of the 44 detected samples; Figure S5: Count of different variants of all compared maternal relatives; Table S1: Comparison of the twice-sequenced libraries; Table S2: MtGenome coverage, observed variants, haplogroup assignment and count of heteroplasmy in the research samples; Table S3: Comparison reports of the 11 kinds of maternal relationships; Table S4. Comparison reports of the 11 kinds of maternal relationships.
