# Supplementary material for: Whole Mitochondrial Genome Detection and Analysis of Two- to Four-Generation Maternal Pedigrees Using a New Massively Parallel Sequencing Panel
Source: Genes (Basel). 2023 Apr 14;14(4):912. doi: 10.3390/genes14040912 (PMC10137955; doi:10.3390/genes14040912)
Supplement: Supplementary file 1 [file genes-14-00912-s001.zip › genes-2316997-supplementary.pdf]

Supplementary Figure S1. Family tree of the four-generation and three-generation pedigree. a. The four-generation pedigree. b. The three-generation pedigree. Individuals without sample ID were not included in this study.

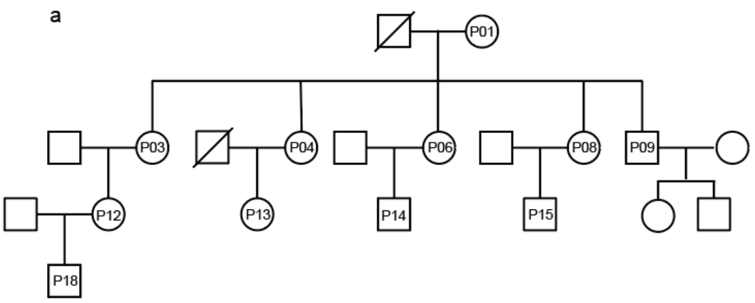

Supplementary Figure S2. Relative read depth of three negative controls and HL60.

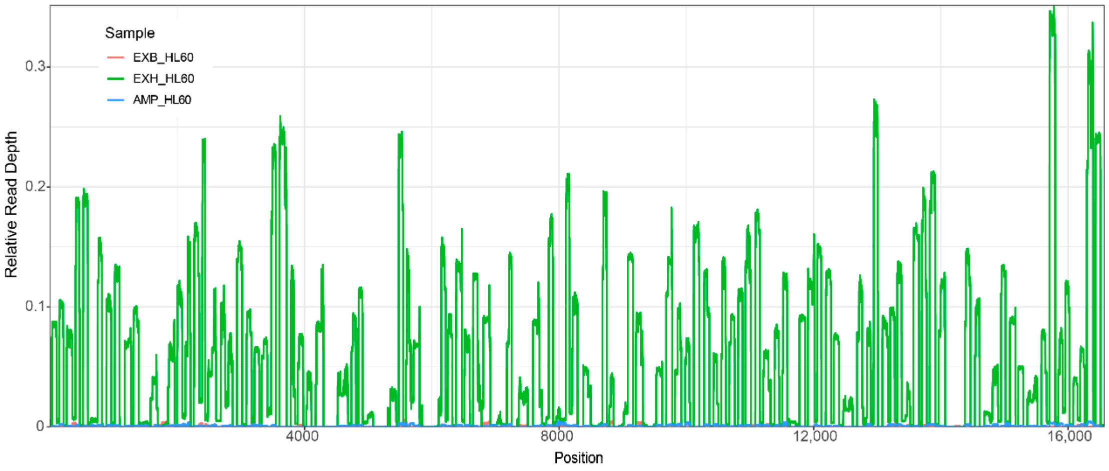

Supplementary Figure S3. Total read depth and mtGenome coverage of the twice-sequenced libraries.

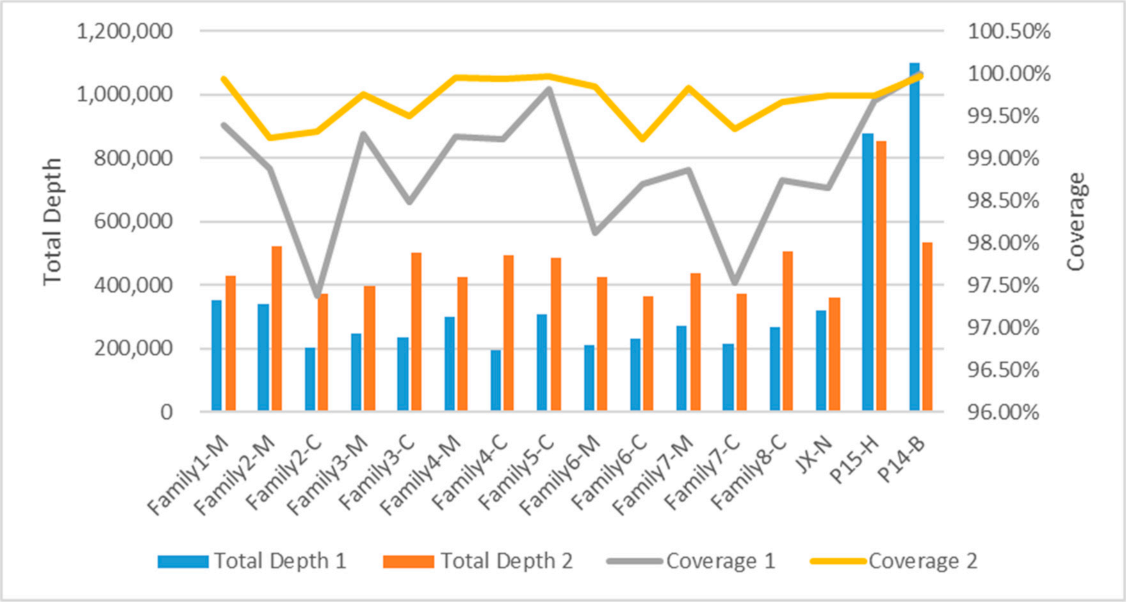

Supplementary Figure S4. Distribution of PHP count of the 44 detected samples.

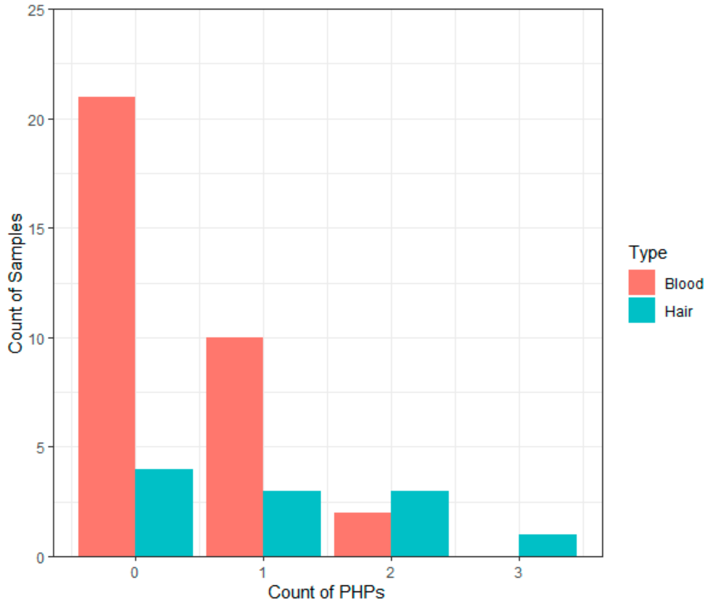

Supplementary Figure S5. Count of different variants of all compared maternal relatives.
a. Count of different variants of full-siblings.
b. Count of different variants of maternal grandmother and maternal grandson/granddaughter.
c. Count of different variants of maternal aunt and maternal nephew/niece.
d. Count of different variants of maternal uncle and maternal nephew/niece.
e. Count of different variants of maternal first cousins.
f. Count of different variants of maternal great grandmother and maternal great grandson.
g. Count of different variants of maternal grandaunt and maternal grandnephew.
h. Count of different variants of maternal granduncle and maternal grandnephew.
i. Count of different variants of maternal cousin-aunt and maternal cousin-nephew.
j. Count of different variants of maternal cousin-uncle and maternal cousin-nephew.

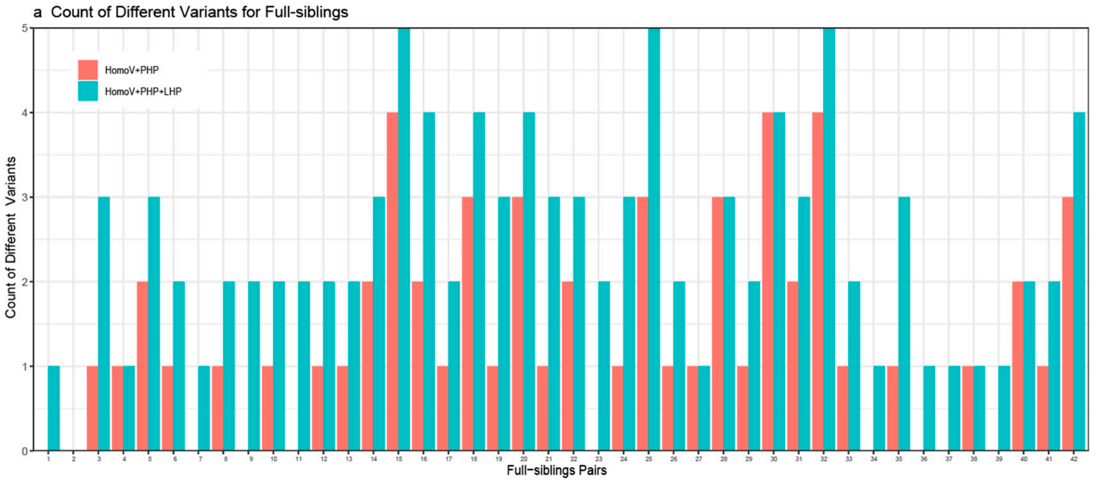

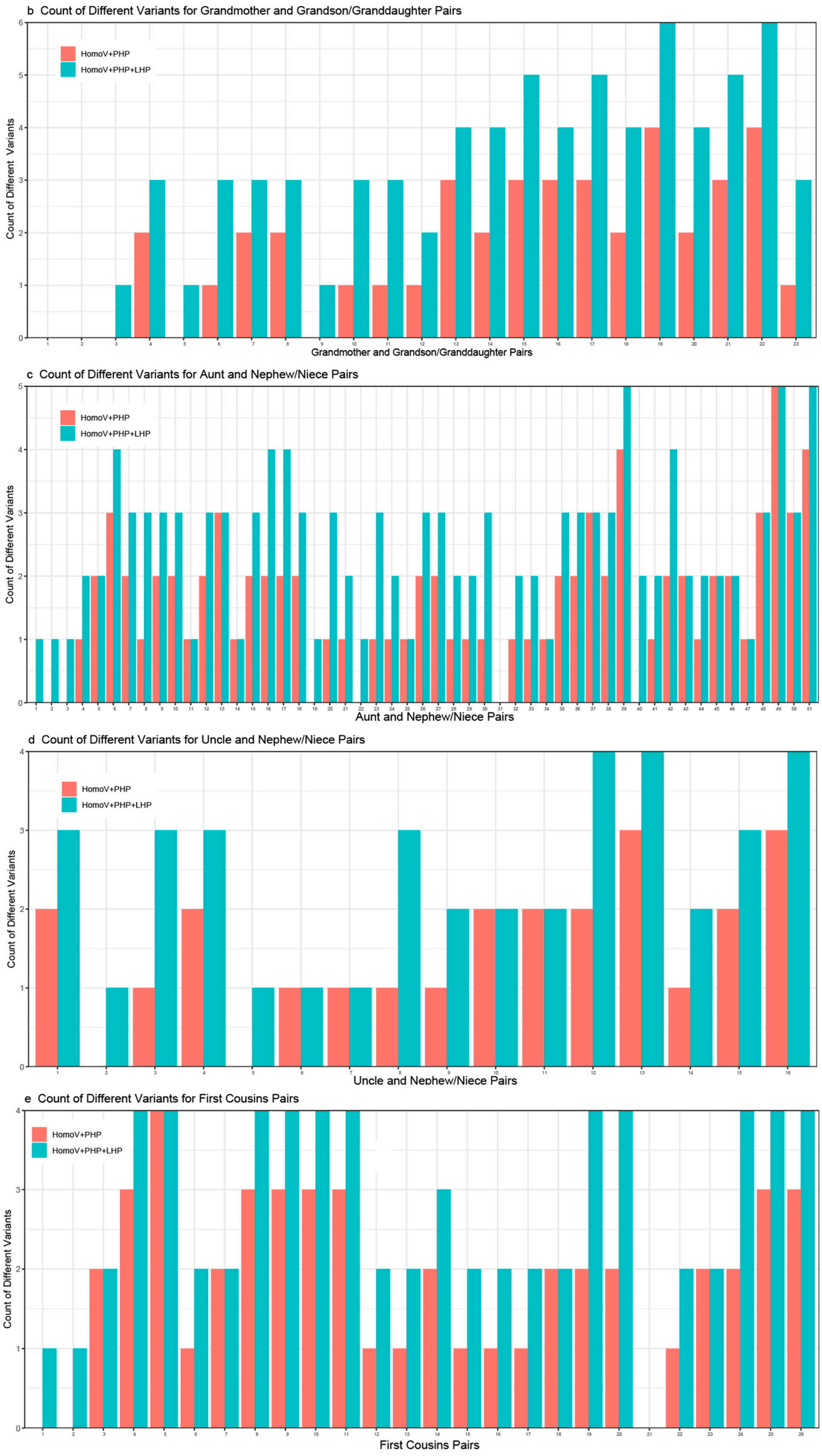
b Count of Different Variants for Grandmother and Grandson/Granddaughter Pairs
HomoV+PHP
HomoV+PHP+LHP
Count of Different Variants
Grandmother and Grandson/Granddaughter Pairs
c Count of Different Variants for Aunt and Nephew/Niece Pairs
HomoV+PHP
HomoV+PHP+LHP
Count of Different Variants
Aunt and Nephew/Niece Pairs
d Count of Different Variants for Uncle and Nephew/Niece Pairs
HomoV+PHP
HomoV+PHP+LHP
Count of Different Variants
Uncle and Nephew/Niece Pairs
e Count of Different Variants for First Cousins Pairs
HomoV+PHP
HomoV+PHP+LHP
Count of Different Variants
First Cousins Pairs

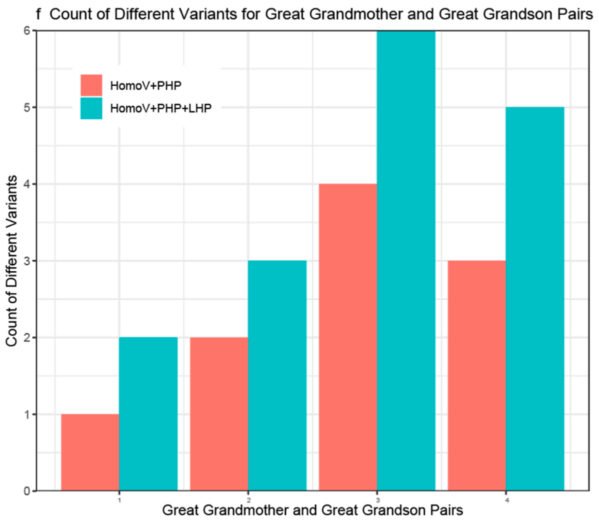
f Count of Different Variants for Great Grandmother and Great Grandson Pairs
HomoV+PHP
HomoV+PHP+LHP
Count of Different Variants
Great Grandmother and Great Grandson Pairs

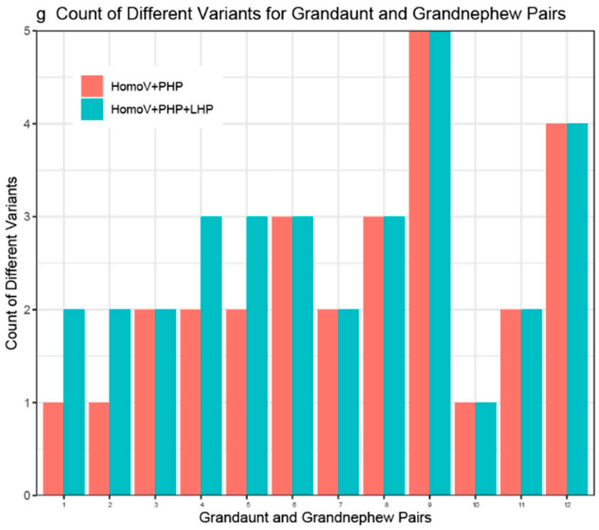
g Count of Different Variants for Grandaunt and Grandnephew Pairs
HomoV+PHP
HomoV+PHP+LHP
Count of Different Variants
Grandaunt and Grandnephew Pairs

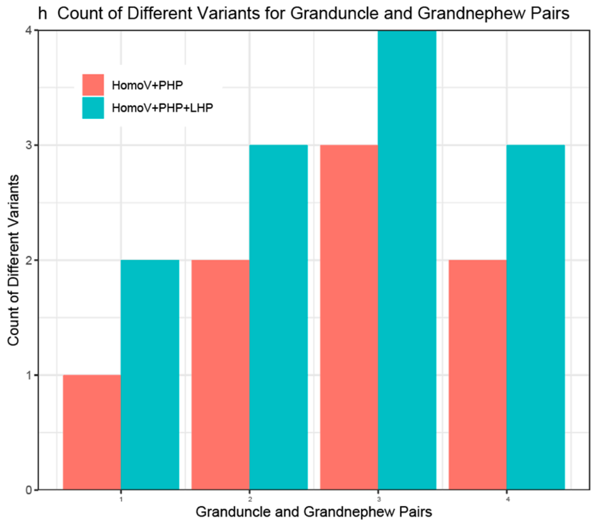
h Count of Different Variants for Granduncle and Grandnephew Pairs
HomoV+PHP
HomoV+PHP+LHP
Count of Different Variants
Granduncle and Grandnephew Pairs

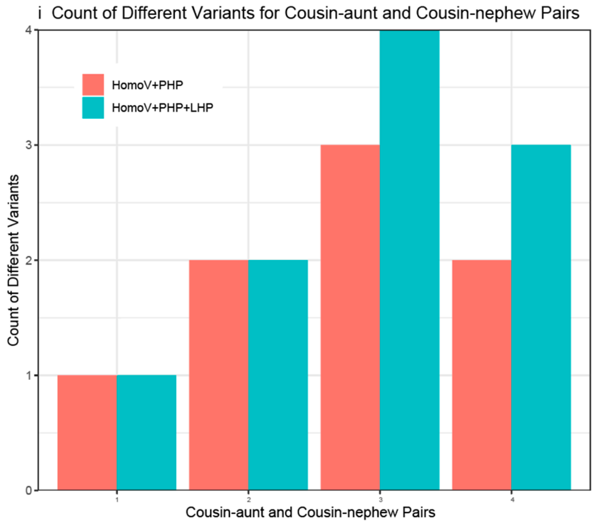
i Count of Different Variants for Cousin-aunt and Cousin-nephew Pairs
HomoV+PHP
HomoV+PHP+LHP
Count of Different Variants
Cousin-aunt and Cousin-nephew Pairs

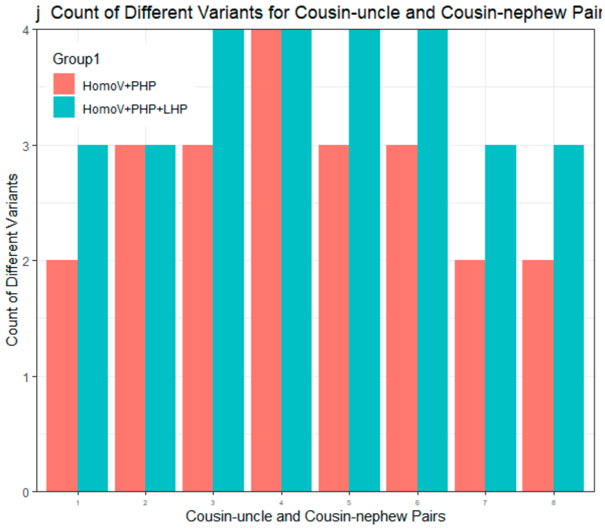
j Count of Different Variants for Cousin-uncle and Cousin-nephew Pair
Group1
HomoV+PHP
HomoV+PHP+LHP
Count of Different Variants
Cousin-uncle and Cousin-nephew Pairs

Supplementary Table S1. Comparison of the twice-sequenced libraries.

	Depth Difference[a]	Coverage Difference	Count of 'No Call' Difference	Difference between variants	Details of Variant Difference (first & second)
Family1-M	74300	0.54%	-90	No	/
Family2-M	183964	0.36%	-60	No	/
Family2-C	169177	1.94%	-321	Yes	309.1C & 309.1c: 88.6% (263 reads) base call C and 11.4% (34 reads, below AT) reference reads present at position 309.1 in the first run, 90.4% (572 reads) base call C and 9.6% (61 reads, above AT) reference reads present at position 309.1 in the second run.
Family3-M	148470	0.48%	-79	Yes	309.1C & 309.1c: 88.6% (263 reads) base call C and 11.4% (34 reads, below AT) reference reads present at position 309.1 in the first run, 81.3% (244 reads) base call C and 18.7% (56 reads, above AT) reference reads present at position 309.1 in the second run.
Family3-C	267576	1.01%	-168	No	/
Family4-M	127460	0.70%	-116	Yes	961C & 961c: 100% (930 reads) base call C present at position 961 in the first run, 93.6% (1158 reads) base call C and 6.4% (79 reads, above AT) deletion present at position 961 in the second run.

Family4-C	299532	0.71%	-118	Yes	309.2C & 309.2c, 965.1c & 965.2c: 67.6% (75 reads) base call C and 32.4% (36 reads, below AT) reference reads present at position 309.2 in the first run, 68.1% (141 reads) base call C and 31.9% (66 reads, above AT) reference reads present at position 309.2 in the second run; no base call present at 965.2 in the first run, 17.9% (71 reads, above AT) base call C and 82.1% (326 reads) reference reads present at position965.2 in the second run.
Family5-C	180251	0.15%	-25	No	/
Family6-M	216423	1.73%	-286	Yes	N[b] & 309.1C, N & 315.1C: no reads was detected at the amplicon where 309 and 315 located in the first run, 89.5% (51 reads, above AT) base call C present at position 309.1 and 96.4% (53 reads, above AT) base call C present at position 315.1 in the second run.
Family6-C	133173	0.54%	-89	Yes	309.1C & N: 100% (46 reads, above AT) base call C present at position 309.1 in the first run, no reads were detected in the second run.
Family7-M	166767	0.97%	-161	Yes	8271DEL & 8271a, N & 16193.1c: 100% (10985 reads) base deletion present at position 8271 in the first run, 89.7% (17443 reads) base deletion and 10.9% (2143 reads, above AT) base call A present at position 8271 in the second run; no reads were detected at position 16193.1 in the first run, 10.9% (62 reads, above AT) base call A and 89.1% (508

					reads) reference reads present at position 16193.1 in the second run.
Family7-C	157583	1.82%	-302	Yes	N & 7364G, 8271DEL & 8271a, 16182C & 16182c, N & 16193.1c, N & 16217C: no reads was detected at position 7364 in the first run, 100% (90 reads, above AT) base call G present at position7364 in the second run; 100% (7974 reads) base deletion present at position 8271 in the first run, 93.3% (12482 reads) base deletion and 6.7% (902 reads, above AT) base call A present at position 8271 in the second run; 100% (205 reads) base call C present at position 16182 in the first run, 85.2% (387 reads) base call C and 14.8% (67 reads, above AT) deletion present at position 16182 in the second run; no reads was detected at position 16193.1 in the first run, 14.4% (66 reads, above AT) base call A and 85.6% (393 reads) reference reads present at position 16193.1 in the second run; no reads was detected at position 162117 in the first run, 100% (74 reads, above AT) base call C present at position16217 in the second run.
Family8-C	238623	0.91%	-151	No	/
JX-N	40738	1.10%	-182	No	/
P15-H	-24703	0.07%	6	No	/
P14-B	-564921	-0.04%	-11	Yes	965.3c & 965.2c: 10.3% (45 reads, above AT) base call C and 89.7% (390 reads) reference reads

present at position965.3 in the first run, no base call present at 965.3 in the second run.

[a] Difference = data of the second run - data of the first run;
[b] 'N' represents no base call at this position.

Supplementary Table S2. MtGenome coverage, observed variants, haplogroup assignment and count of heteroplasmy in the research samples.

Sample ID	mtGenome Coverage	Observed Variants	Haplogroup	Super Haplogroup	Count of PHP/LHP
Family1-M	99.93%	73G 152Y 194T 199C 263G 315.1C 489C 523a524c 750G 1382C 1438G 2706G 3010A 4769G 4883T 5178A 5616G 7028T 8020A 8414T 8701G 8860G 8964T 9296T 9540C 9824A 10398G 10400T 10873C 11719A 12705T 14668T 14766T 14783C 15043A 15301A 15326G 16094C 16111T 16187T 16223T 16362C 16519C	D4b2b	M	1/1
Family1-C	99.55%	73G 152Y 194T 199C 263G 315.1C 489C 523a524c 750G 1382C 1438G 2706G 3010A 4769G 4883T 5178A 5616G 7028T 8020A 8414T 8701G 8860G 8964T 9296T 9540C 9824A 10398G 10400T 10873C 11719A 12705T 14668T 14766T 14783C 15043A 15301A 15326G 16094C 16111T 16187T 16223T 16362C 16519C	D4b2b	M	1/1
Family2-M	99.24%	73G 150T 199C 204C 263G 309.1c 315.1C 489C 750G 1438G 2706G 3736A 4164G 4769G 5351G 5460A 6455T 6680C 7028T 7444A 7684C 7853A 8701G 8860G 9540C 9824C 10397G 10398G 10400T 10873C 11719A 12405T 12705T 12811C 14766T 14783C 15043A 15301A 15326G 16129A 16192T 16223T 16297C 16362C 16400T	M7b1a1h	M	0/1
Family2-C	99.31%	73G 150T 199C 204C 263G 309.1c 315.1C 489C 750G 1438G 2706G 3736A 4164G 4769G 5351G 5460A 6455T 6680C 7028T 7444A 7684C	M7b1a1h	M	0/1

		7853A 8701G 8860G 9540C 9824C 10397G 10398G 10400T 10873C 11719A 12405T 12705T 12811C 14766T 14783C 15043A 15301A 15326G 16129A 16192T 16223T 16297C 16362C 16400T			
Family3-M	99.76%	73G 228A 249DEL 263G 309.1c 315.1C 523a524c 548T 750G 1438G 2706G 3645C 3970T 4086T 4769G 6392C 6962A 7028T 8479G 8860G 9053A 9548A 10211T 10310A 10609C 11719A 12361R 12406A 12882T 13759A 13928C 14325C 14766T 15326G 16129A 16162G 16172C 16274A 16304C 16311C 16519C	F1a1c	N	1/2
Family3-C	99.50%	73G 228A 249DEL 263G 309.1C 315.1C 523a524c 548T 750G 1438G 2706G 3645C 3970T 4086T 4769G 6392C 6962A 7028T 8479G 8860G 9053A 9548A 10211T 10310A 10609C 11719A 12406A 12882T 13759A 13928C 14325C 14766T 15326G 16129A 16162G 16172C 16274A 16304C 16311C 16519C	F1a1c	N	0/1
Family4-M	99.95%	73G 150T 263G 309.1C 309.2c 315.1C 750G 960.1C 960.2C 961C 1438G 2706G 4769G 5231A 5417A 5773A 7028T 8860G 11719A 12358G 12372A 12705T 12950G 14766T 15067C 15326G 16223T 16257A 16261T	N9a2	N	0/2
Family4-C	99.93%	73G 150T 263G 309.1C 309.2c 315.1C 750G 960.1C 960.2C 961C 1438G 2706G 4769G 5231A 5417A 5773A 7028T 8860G 11719A 12358G 12372A 12705T 12950G 14766T 15067C 15326G 16223T 16257A 16261T	N9a2	N	0/2
Family5-M	99.95%	73G 152C 194T 263G 315.1C 489C 523a524c 750G 1382C 1438G 2706G 3010A 4769G 4883T 5178A 7028T 8020A 8414T 8577G 8701G 8860G 8964T 9296T 9540C 9824A 10398G 10400T 10873C 11719A 12358G 12705T 14668T 14766T 14783C 15043A 15301A 15326G 16172C 16362C 16519C	D4b2b2b	M	0/1

Family5-C	99.96%	73G 152C 194T 263G 315.1C 489C 523a524c 750G 1382C 1438G 2706G 3010A 4769G 4883T 5178A 7028T 8020A 8414T 8577G 8701G 8860G 8964T 9296T 9540C 9824A 10398G 10400T 10873C 11719A 12358G 12705T 14668T 14766T 14783C 15043A 15301A 15326G 16172C 16362C 16519C	D4b2b2b	M	0/1
Family6-M	99.84%	73G 257G 263G 309.1C 315.1C 489C 750G 856G 1438G 2706G 2766T 3010A 4769G 4883T 5178A 6986G 7028T 8414T 8701G 8860G 9540C 10042G 10398G 10400T 10873C 11719A 12705T 14668T 14766T 14783C 15043A 15301A 15326G 15704T 16224C 16245T 16292T 16362C 16519C	D4c1b	M	0/0
Family6-C	98.69%	73G 257G 263G 309.1C 315.1C 489C 750G 856G 1438G 2706G 2766T 3010A 4769G 4883T 5178A 6986G 7028T 8414T 8701G 8860G 9540C 10042G 10398G 10400T 10873C 11719A 12705T 14668T 14766T 14783C 15043A 15301A 15326G 15704T 16224C 16245T 16292T 16362C 16519C	D4c1b	M	0/0
Family7-M	99.82%	73G 146C 195C 263G 315.1C 523a524c 750G 1438G 2706G 4769G 5093C 7028T 7364G 8271-8279DEL 8860G 8952C 11719A 12028C 13269G 14766T 15115Y 15326G 16129A 16182C 16183C 16189C 16193.1c 16217C 16261T	B4h	N	1/3
Family7-C	99.35%	73G 146C 195C 263G 315.1C 523a524c 750G 1438G 2706G 4769G 5093C 7028T 7364G 8271-8279DEL 8860G 8952C 11719A 12028C 13269G 14766T 15326G 16129A 16182C 16183C 16189C 16193.1c 16217C 16261T	B4h	N	0/3
Family8-M	99.34%	73G 189G 249DEL 263G 309.1C 315.1C 750G 1005C 1438G 1824C 2706G 3386Y 3970T 4769G 6392C 7028T 7828G 8860G 10310A 10535C 10586A 11150A 11719A 12338C 13708A 13722G 13928C	F2c1	N	1/1

		14766T 15326G 15714T 16183C 16189C 16193.1c 16304C 16355T 16519C			
Family8-C	99.66%	73G 189G 249DEL 263G 309.1C 315.1C 750G 1005C 1438G 1824C 2706G 3386Y 3970T 4769G 6392C 7028T 7828G 8860G 10310A 10535C 10586A 11150A 11719A 12338C 13708A 13722G 13928C 14766T 15326G 15714T 16183C 16189C 16193.1c 16304C 16355T 16519C	F2c1	N	1/1
JX-N	99.74%	73G 152C 263G 309.1c 315.1C 489C 750G 1438G 2706G 4715G 4769G 4958G 5100T 5402G 6179A 7028T 7196A 8584A 8684T 8701G 8860G 9540C 10398G 10400T 10873C 11719A 12705T 13488C 14470C 14766T 14783C 15043A 15301A 15326G 15487T 16184T 16223T 16293G 16298C 16319A	M8a3	M	0/1
JX-M1	99.94%	73G 152C 263G 309.1c 315.1C 489C 750G 1438G 2706G 4715G 4769G 4958G 5100T 5402G 6179A 7028T 7196A 8584A 8684T 8701G 8860G 9540C 10398G 10400T 10873C 11719A 12705T 13488C 14470C 14766T 14783C 15043A 15301A 15326G 15487T 16184T 16223T 16293G 16298C 16319A	M8a3	M	0/1
JX-C1	99.70%	73G 152C 263G 309.1c 315.1C 489C 750G 1438G 2706G 4715G 4769G 4958G 5100T 5402G 6179A 7028T 7196A 8584A 8684T 8701G 8860G 9540C 10398G 10400T 10873C 11719A 12705T 13488C 14470C 14766T 14783C 15043A 15301A 15326G 15487T 16184T 16223T 16293G 16298C 16319A	M8a3	M	0/1
JX-C2	99.98%	73G 152C 263G 309.1c 315.1C 489C 750G 1438G 2706G 4715G 4769G 4958G 5100T 5402G 6179A 7028T 7196A 8584A 8684T 8701G 8860G 9540C 10398G 10400T 10873C 11719A 12705T 13488C	M8a3	M	0/1

		14470C 14766T 14783C 15043A 15301A 15326G 15487T 16184T 16223T 16293G 16298C 16319A			
JX-M2	99.99%	73G 152C 263G 309.1C 315.1C 489C 750G 1438G 2706G 4715G 4769G 4958G 5100T 5402G 6179A 7028T 7196A 8584A 8684T 8701G 8860G 9540C 10398G 10400T 10873C 11719A 12705T 13488C 14470C 14766T 14783C 15043A 15301A 15326G 15487T 16184T 16223T 16293G 16298C 16319A	M8a3	M	0/0
JX-C3	99.93%	73G 152C 263G 309.1C 315.1C 489C 750G 1438G 2706G 4715G 4769G 4958G 5100T 5402G 6179A 7028T 7196A 8584A 8684T 8701G 8860G 9540C 10398G 10400T 10873C 11719A 12705T 13488C 14470C 14766T 14783C 15043A 15301A 15326G 15487T 16184T 16223T 16293G 16298C 16319A	M8a3	M	0/0
P01-B	100.00%	73G 235G 263G 309.1C 315.1C 318C 523a524c 663G 750G 960.1C 960.2C 961C 1415A 1438G 1736G 2706G 4248C 4769G 4824G 7028T 7657C 8563G 8794T 8860G 11536T 11719A 12705T 13260C 14766T 14854T 15326G 16184T 16223T 16290T 16319A 16519C	A5b	N	0/2
P01-H	100.00%	73G 235G 251R 263G 315.1C 318C 523a524c 663G 750G 960.1C 960.2C 961C 1415A 1438G 1736G 2706G 4248C 4769G 4824G 7028T 7657C 8563G 8794T 8860G 11118Y 11536T 11719A 12705T 13260C 14766T 14854T 15326G 16184T 16223T 16290T 16319A 16519C	A5b	N	2/2
P03-B	100.00%	73G 235G 263G 309.1C 315.1C 318C 523a524c 663G 750G 960.1C 960.2C 960.3C 961C 1415A 1438G 1736G 2706G 4248C 4769G 4824G 7028T 7657C 8563G 8794T 8860G 11536T 11719A 12705T 13260C 13683M 14766T 14854T 15326G 16184T 16223T 16290T 16319A 16519C	A5b	N	1/2

P03-H	100.00%	73G 235G 263G 309.1c 315.1C 318C 523a524c 663G 750G 960.1C 960.2C 960.3C 961C 1415A 1438G 1736G 2706G 4248C 4769G 4824G 7028T 7657C 8563G 8794T 8860G 11536T 11719A 12705T 13260C 14766T 14854T 15326G 16184T 16223T 16290T 16319A 16519C	A5b	N	0/3
P04-B	100.00%	73G 235G 263G 309.1C 315.1C 318C 523a524c 663G 750G 960.1C 960.2C 961C 1415A 1438G 1736G 2706G 4248C 4769G 4824G 7028T 7657C 8563G 8794T 8860G 11536T 11719A 12705T 13260C 14766T 14854T 15326G 16184T 16223T 16290T 16319A 16519C	A5b	N	0/2
P04-H	100.00%	73G 235G 263G 309.1C 315.1C 318C 523a524c 663G 750G 960.1C 960.2C 961C1415A 1438G 1736G 2706G 4248C 4769G 4824G 7028T 7657C 8563G 8794T 8860G 11536T 11719A 12705T 13260C 14766T 14854T 15326G 16184T 16223T 16290T 16319A 16519C	A5b	N	0/2
P06-B	100.00%	73G 235G 263G 309.1C 315.1C 318C 523a524c 663G 750G 960.1C 960.2C 960.3C 961C 1415A 1438G 1736G 2706G 4248C 4769G 4824G 7028T 7657C 8563G 8794T 8860G 11536T 11719A 12705T 13260C 14766T 14854T 15326G 16184T 16223T 16290T 16319A 16519C	A5b	N	0/2
P06-H	100.00%	73G 235G 263G 309.1C 315.1C 318C 523a524c 663G 750G 960.1C 960.2C 961C 1415A 1438G 1736G 2706G 4248C 4475C 4769G 4824G 7028T 7657C 8563G 8794T 8860G 11536T 11719A 12705T 13260C 14766T 14854T 15326G 16184T 16223T 16290T 16319A 16519C	A5b	N	0/2
P08-B	100.00%	73G 235G 263G 309.1C 315.1C 318C 523a524c 663G 750G 960.1C 960.2C 961C 1415A 1438G 1736G 2706G 4248C 4769G 4824G 7028T 7657C 8563G 8794T 8860G 9083Y 11536T 11719A 12705T 13260C 14766T 14854T 15326G 16184T 16223T 16290T 16319A 16519C	A5b	N	1/2

P08-H	100.00%	73G 235G 263G 309.1C 315.1C 318C 523a524c 663G 750G 960.1C 960.2C 961C 1415A 1438G 1736G 2706G 4248C 4769G 4824G 5843R 7028T 7657C 8563G 8794T 8860G 11536T 11719A 12705T 13260C 13269R 13679M 14766T 14854T 15326G 16184T 16223T 16290T 16319A 16519C	A5b	N	3/2
P09-B	100.00%	73G 235G 263G 309.1c 315.1C 318C 523a524c 663G 750G 960.1C 960.2C 961C 1415A 1438G 1736G 2706G 4248C 4769G 4824G 7028T 7657C 8563G 8794T 8860G 11536T 11719A 12705T 13260C 14766T 14854T 15326G 16184T 16223T 16290T 16319A 16519C	A5b	N	0/3
P09-H	100.00%	73G 235G 263G 309.1c 315.1C 318C 523a524c 663G 750G 960.1C 960.2C 961C 1415A 1438G 1736G 2706G 3290Y 4248C 4769G 4776S 4824G 7028T 7657C 8563G 8794T 8860G 11536T 11719A 12705T 13260C 14766T 14854T 15326G 16184T 16223T 16290T 16319A 16519C	A5b	N	2/3
P12-B	100.00%	73G 235G 263G 309.1C 315.1C 318C 523a524c 663G 750G 960.1C 960.2C 961C 1415A 1438G 1736G 2706G 4248C 4769G 4824G 7028T 7657C 8563G 8794T 8860G 11536T 11719A 12705T 13260C 13678M 14215Y 14766T 14854T 15326G 16184T 16223T 16290T 16319A 16519C	A5b	N	2/2
P12-H	100.00%	73G 235G 263G 309.1C 315.1C 318C 523a524c 663G 750G 960.1C 960.2C 961C 1415A 1438G 1736G 2706G 4248C 4769G 4824G 7028T 7657C 8563G 8794T 8860G 11536T 11719A 12425.1a 12705T 13260C 14766T 14854T 15326G 16184T 16223T 16290T 16319A 16519C	A5b	N	0/3
P13-B	100.00%	73G 235G 263G 309.1C 315.1C 318C 523a524c 663G 750G 960.1C 960.2C 961C 1415A 1438G 1736G 2706G 4248C 4769G 4824G 7028T	A5b	N	0/2

		7657C 8563G 8794T 8860G 11536T 11719A 12705T 13260C 14766T 14854T 15326G 16184T 16223T 16290T 16319A 16519C			
P13-H	100.00%	73G 235G 263G 309.1c 315.1C 318C 523a524c 663G 750G 960.1C 960.2C 961C 1415A 1438G 1736G 2706G 4248C 4769G 4824G 7028T 7657C 8563G 8794T 8860G 11536T 11719A 12705T 13260C 13679M 14766T 14854T 15326G 16184T 16223T 16290T 16319A 16519C	A5b	N	1/3
P14-B	100.00%	73G 235G 263G 309.1C 315.1C 318C 523a524c 663G 750G 960.1C 960.2C 960.3C 961C 1415A 1438G 1736G 2706G 4248C 4769G 4824G 7028T 7657C 8563G 8794T 8860G 11536T 11719A 12705T 13260C 13683S 14766T 14854T 15326G 16184T 16223T 16290T 16319A 16519C	A5b	N	1/2
P14-H	100.00%	73G 235G 263G 309.1c 315.1C 318C 523a524c 663G 750G 960.1C 960.2C 961C 1227R 1415A 1438G 1736G 2706G 4248C 4769G 4824G 7028T 7657C 8563G 8794T 8860G 11536T 11719A 12705T 13260C 14766T 14854T 15326G 16184T 16223T 16290T 16319A 16519C	A5b	N	1/3
P15-B	100.00%	73G 235G 263G 309.1C 315.1C 318C 523a524c 663G 750G 960.1C 960.2C 961C 1415A 1438G 1736G 2706G 4248C 4769G 4824G 7028T 7657C 8563G 8794T 8860G 9083Y 11536T 11719A 12705T 13260C 14766T 14854T 15326G 16154Y 16184T 16223T 16290T 16319A 16519C	A5b	N	2/2
P15-H	99.74%	73G 235G 263G 309.1C 315.1C 318C 523a524c 663G 750G 961C 1415A 1438G 1606R 1736G 2706G 4248C 4769G 4824G 7028T 7657C 8563G 8794T 8860G 11536T 11719A 12705T 13260C 14766T 14854T 15326G 16184T 16223T 16290T 16319A 16519C	A5b	N	1/1
P18-B	100.00%	73G 235G 263G 309.1C 315.1C 318C 523a524c 663G 750G 960.1C 960.2C 961C 1415A 1438G 1736G 2706G 4248C 4769G 4824G 7028T	A5b	N	1/2

		7657C 8563G 8794T 8860G 11536T 11719A 12705T 13260C 14766T 14854T 15326G 16184T 16223T 16278Y 16290T 16319A 16519C			
P18-H	100.00%	73G 235G 263G 309.1C 315.1C 318C 523a524c 663G 750G 960.1C 960.2C 961C 1415A 1438G 1736G 2706G 4248C 4769G 4824G 5591R 7028T 7657C 8563G 8794T 8860G 11536T 11719A 12705T 13260C 14215Y 14766T 14854T 15326G 16184T 16223T 16290T 16319A 16519C	A5b	N	2/2

Supplementary Table S3. Different variations of the HL60 samples in different studies.

Position	This study	Validation of ForenSeq mtGenome Kit [42]	Lot-to-lot study of control DNA[54]	Certificate of SRM2392-I [55]
2445	T2445 (100% T)	2445Y	2445Y	T2445
4821	4821R (7.4% A)	G4821	G4821	G4821
12071	T12071 (1.1% C)	12071Y	12071Y	12071Y

Supplementary Table S4. Comparison reports of the 11 kinds of maternal relationships.

a. Homoplasmic variants only

Relationship	Cannot Exclude	Inconclusive	Exclusion	%Cannot Exclude	%Inconclusive	%Exclusion
mother and offspring	49	4	0	92.45%	7.55%	0.00%
full-siblings	34	8	0	80.95%	19.05%	0.00%
maternal grandmother and maternal grandson/granddaughter	23	0	0	100.00%	0.00%	0.00%
maternal aunt and maternal nephew/niece	45	6	0	88.24%	11.76%	0.00%

maternal uncle and maternal nephew/niece	16	0	0	100.00%	0.00%	0.00%
maternal first cousins	26	0	0	100.00%	0.00%	0.00%
maternal great grandmother and maternal great grandson	4	0	0	100.00%	0.00%	0.00%
maternal grandaunt and maternal grandnephew	10	2	0	83.33%	16.67%	0.00%
maternal granduncle and maternal grandnephew	4	0	0	100.00%	0.00%	0.00%
maternal cousin-aunt and maternal cousin-nephew	4	0	0	100.00%	0.00%	0.00%
maternal cousin-uncle and maternal cousin-nephew	8	0	0	100.00%	0.00%	0.00%

b. Homoplasmic variants and PHPs

Relationship	Cannot Exclude	Inconclusive	Exclusion	%Cannot Exclude	%Inconclusive	%Exclusion	%Exclusion Compared to HomoV[a] only
mother and offspring	20	12	21	37.74%	22.64%	39.62%	39.62%
full-siblings	10	18	14	23.81%	42.86%	33.33%	33.33%
maternal grandmother and maternal grandson/granddaughter	5	5	13	21.74%	21.74%	56.52%	56.52%
maternal aunt and maternal nephew/niece	7	18	26	13.73%	35.29%	50.98%	50.98%
maternal uncle and maternal nephew/niece	2	6	8	12.50%	37.50%	50.00%	50.00%

maternal first cousins	3	7	16	11.54%	26.92%	61.54%	61.54%
maternal great grandmother and maternal great grandson	0	1	3	0.00%	25.00%	75.00%	75.00%
maternal grandaunt and maternal grandnephew	0	3	9	0.00%	25.00%	75.00%	75.00%
maternal granduncle and maternal grandnephew	0	1	3	0.00%	25.00%	75.00%	75.00%
maternal cousin-aunt and maternal cousin-nephew	0	1	3	0.00%	25.00%	75.00%	75.00%
maternal cousin-uncle and maternal cousin-nephew	0	0	8	0.00%	0.00%	100.00%	100.00%

c. Homoplasmic variants, PHPs and LHPs

Relationship	Cannot Exclude	Inconclusive	Exclusion	%Cannot Exclude	%Inconclusive	%Exclusion	%Exclusion Compared to HomoV only	%exclusion compared to HomoV+PHP
mother and offspring	11	9	33	20.75%	16.98%	62.26%	62.26%	22.64%
full-siblings	1	9	32	2.38%	21.43%	76.19%	76.19%	42.86%
maternal grandmother and maternal grandson/granddaughter	2	3	18	8.70%	13.04%	78.26%	78.26%	21.74%
maternal aunt and maternal nephew/niece	1	10	40	1.96%	19.61%	78.43%	78.43%	27.45%
maternal uncle and maternal nephew/niece	0	4	12	0.00%	25.00%	75.00%	75.00%	25.00%
maternal first cousins	1	2	23	3.85%	7.69%	88.46%	88.46%	26.92%

maternal great grandmother and maternal great grandson	0	0	4	0.00%	0.00%	100.00%	100.00%	25.00%
maternal grandaunt and maternal grandnephew	0	1	11	0.00%	8.33%	91.67%	91.67%	16.67%
maternal granduncle and maternal grandnephew	0	0	4	0.00%	0.00%	100.00%	100.00%	25.00%
maternal cousin-aunt and maternal cousin-nephew	0	1	3	0.00%	25.00%	75.00%	75.00%	0.00%
maternal cousin-uncle and maternal cousin-nephew	0	0	8	0.00%	0.00%	100.00%	100.00%	0.00%

[a] HomoV: homoplasmic variant.
